# A revision of *Pachyballus* Simon, 1900 and *Peplometus* Simon, 1900 (Araneae, Salticidae, Ballini) with descriptions of new species

**DOI:** 10.3897/zookeys.944.49921

**Published:** 2020-06-30

**Authors:** Wanda Wesołowska, Galina N. Azarkina, Konrad Wiśniewski

**Affiliations:** 1 Department of Biodiversity and Evolutionary Taxonomy, University of Wrocław, Przybyszewskiego 65, 51-148 Wrocław, Poland University of Wrocław Wrocław Poland; 2 Laboratory of Systematics of Invertebrate Animals, Institute of Systematics and Ecology of Animals, Siberian Branch Russian Academy of Sciences, Frunze Street 11, Novosibirsk 630091, Russia Institute of Systematics and Ecology of Animals, Siberian Branch Russian Academy of Sciences Novosibirsk Russia; 3 Institute of Biology and Earth Sciences, Pomeranian University in Słupsk, Arciszewskiego 22b, 76-200 Słupsk, Poland Pomeranian University in Słupsk Słupsk Poland

**Keywords:** Africa, jumping spiders, mimicry, new combination, redescription, synonyms, taxonomy

## Abstract

Two genera from the tribe Ballini (Araneae, Salticidae), *Pachyballus* Simon, 1900 and *Peplometus* Simon, 1900, are remarkable for their resemblance to beetles. Their biology is, however, poorly known and taxonomy has hitherto been rarely analysed. Thirteen species are included in this taxonomic revision of the two genera. Six of them are new to the science: *Pachyballus caelestis***sp. nov.** (♂♀, Congo D.R.), *Pachyballus miniscutulus***sp. nov.** (♂♀, South Africa), *Pachyballus mombasensis***sp. nov.** (♂♀, Kenya), *Pachyballus ornatus***sp. nov.** (♂♀, Congo D.R. and Tanzania), *Peplometus congoensis***sp. nov.** (♂♀, Congo and Congo D.R.), and *Peplometus nimba***sp. nov.** (♂, Guinea). One species (*Pachyballus cordiformis* Berland et Millot, 1941) and a subspecies (*P. flavipes aurantius* Caporiacco, 1949) are recognised as synonyms of *Pachyballus flavipes* Simon, 1910. One new combination is proposed: *Peplometus oyo* (Wesołowska et Russell-Smith, 2011) **comb. nov.** (ex *Pachyballus*). The previously unknown females of *Pachyballus transversus* Simon, 1900 and *Peplometus chlorophthalmus* Simon, 1900, along with the males of *Pachyballus castaneus* Simon, 1900 and *Peplometus biscutellatus* (Simon, 1887) are newly diagnosed and described. Neotypes for *Pachyballus castaneus* and *P. flavipes* are designated. Numerous new data on the distribution are provided here and a key to *Pachyballus* females and to the males of *Peplometus* is presented. Identity of one species remains doubtful, *Pachyballus gambeyi* (Simon, 1880).

## Introduction

Simon (1900) established two African genera, *Pachyballus* and *Peplometus*, for some species that mimic beetles and assigned them to the Balleae group (Simon 1901). Subsequently Petrunkevitch (1928) included them in the subfamily Magoninae. Benjamin (2004) did not study these two genera in his revision of Ballinae. According to the most up to date systematics of the jumping spiders (Maddison 2015), *Pachyballus* and *Peplometus* belong to the tribe Ballini Banks, 1892 in the subfamily Salticinae Blackwall, 1841. Only a few species have been described within these genera up to now, seven as *Pachyballus* (with one subspecies) and two as *Peplometus* (WSC 2019). After our review the species number within the two genera is nine and five respectively.

**Figures 1–6.**
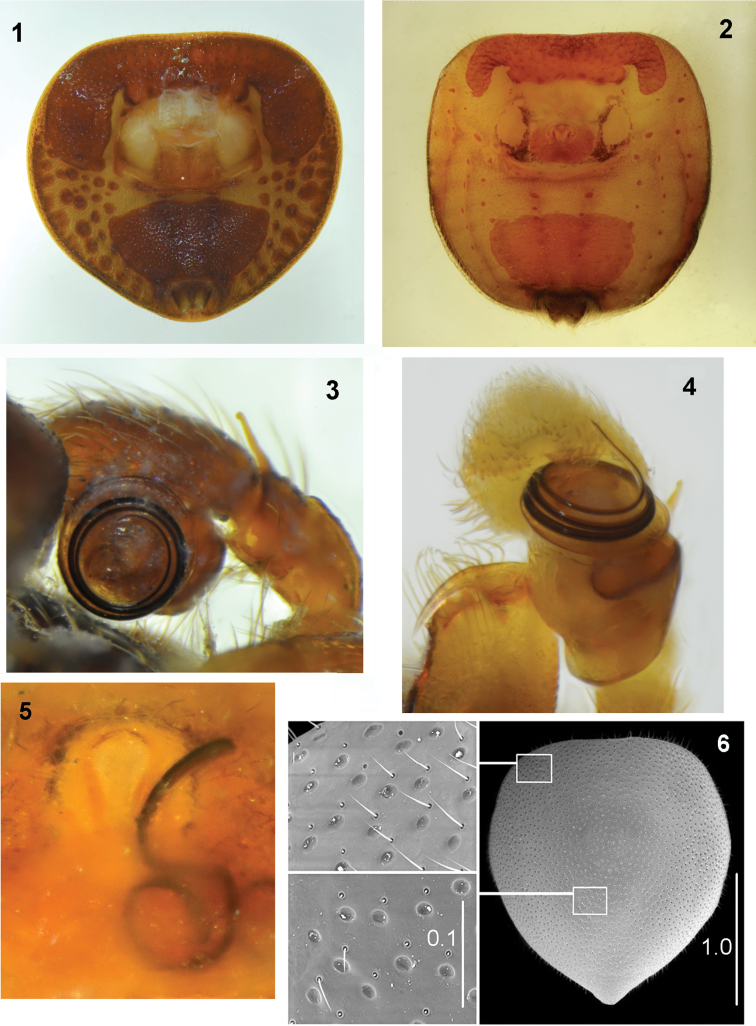
Some morphological characters of *Pachyballus* and *Peplometus***1***Pach. flavipes* (specimen from Gabon), male, ventral abdominal scuta **2***Pach. ornatus* (specimen from Tanzania), female, ventral abdominal scuta **3***Pach. flavipes* (specimen from Congo), embolus **4***Pepl. biscutellatus* (specimen from Ivory Coast), palpal organ in ventral view **5***Pach. flavipes* (specimen from Zimbabwe), epigyne with broken embolus in copulatory opening **6***Pach. castaneus*, pitted integument of abdomen.

*Pachyballus* and *Peplometus* are closely related and share most morphological characters. They are small but robust spiders, with a strongly flattened body (Figs 31, 35, 137), covered with a very hard, sclerotised exoskeleton. The dorsum of their body has a characteristic pitted microsculpture (Fig. 6). The anterior part of the abdomen is covered by posterior edge of the carapace, so that the pedicel is invisible (Figs 99, 172). A putative morphological synapomorphy for these two genera is the presence of characteristic scuta on the ventral surface of their abdomen. This ventral “armament” consists of the two scuta: a narrow one along the anterior margin of the abdomen, which laterally extends backwards, and a posterior trapezoid scutum (Figs 1, 2). There are also numerous minute sclerotised bumps on the ventral side of the abdomen (Fig. 1). These structures, in combination with dorsal strong sclerotisation, make members of these genera among the most heavily armour-plated spiders. Legs are short, and the first pair of male legs is clearly larger than others (Figs 19, 154) and has thickened femora and the tibiae ventrally covered with dense setae.

The conformation of genitalia in both sexes is very similar in all species. Tibial apophysis of the male palp is thin and straight, bulb oval, tegulum has a large posterior lobe, spiralled embolus with more than three coils on the bulb tip (Figs 3, 4). The epigyne has anterior semi-circular depression divided by a median septum (Figs 38, 107), very long copulatory ducts with initial short spiral followed by several loops (Fig. 150), and more or less oval spermathecae (Figs 96, 171). On the sides of the epigynal depression two crevices of unknown role can sometimes be seen (Figs 95, 153). We observed once a broken embolus, which was blocking the copulatory opening (Fig. 5), however we did not notice any other mating plugs.

## Material and methods

Specimens examined it this study are deposited in the following institutions:

**BMNH** British Museum (Natural History) London, United Kingdom

**CAS** California Academy of Sciences, San Francisco, USA

**HNHM** Hungarian Natural History Museum, Budapest, Hungary

**MCZ** Museum of Comparative Zoology, Harvard University, Cambridge, USA

**MEU** Museum of Evolution, Uppsala University, Sweden

**MNHN** National Museum of Natural History, Paris, France

**MRAC** Royal Museum for Central Africa, Tervuren, Belgium

**NCA** National Collection of Arachnida, Pretoria, South Africa

**NMBA** National Museum, Bloemfontein, South Africa

**NHRS** Naturhistoriska Riksmuseet, Stockholm, Sweden

**NMZ** Natural History Museum, Bulawayo, Zimbabwe

**SMF** Senckenberg Natural History Museum, Frankfurt, Germany

**UCZM** Zoological Museum, University of Copenhagen, Denmark

The specimens were examined in 70% ethanol. The epigynes were macerated in cold 5% KOH for 24 hours, dehydrated with absolute ethanol, cleared in xylene and put in clove oil in a temporary microscope slides. A reticular eyepiece attached to a stereomicroscope was used for drawing. After examination and reverting the above described sequence, the female genitalia were placed to microvials and stored with specimens. Specimens were measured as in Metzner (1999); all dimensions are given in millimetres. A Nikon Coolpix 8400 or Canon EOS 550D mounted on the stereomicroscope was used to take digital photos, which were stacked using Helicon Focus image stacking software. Male-female matching was based both on the co-occurrence of specimens and morphological similarities between the sexes (e.g., shape and colouration of body). SEM microphotographs were taken with SEM Hitachi TM–1000. The photographed parts were dried, and then mounted on an adhesive specimen stub. The maps were prepared using DIVA-GIS.

## Distinguishing genera

Due to numerous similarities of *Pachyballus* and *Peplometus*, many collectors have failed to distinguish the two genera and labelled the specimens simply as *Pachyballus*. All previously undetermined specimens analysed in this work had been assigned to *Pachyballus* before and mostly determined only to the genus level. *Peplometus* after the work by Simon (1901) was only found by Berland and Millot (1941) and mentioned by Bodner and Maddison (2012; for specimen from Ghana used in molecular phylogenetic analysis).

**Figures 7, 8.**
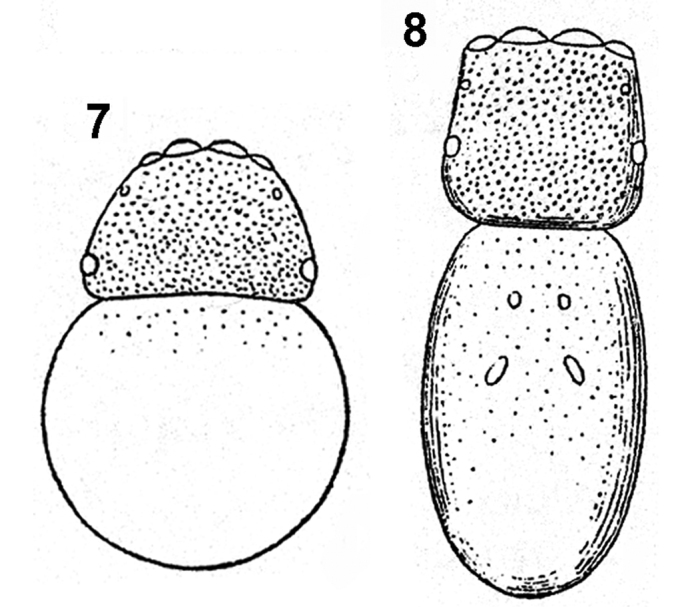
Simon’s drawings of body shape. **7***Pachyballus***8***Peplometus* (from Simon 1901).

Simon (1901) differentiated the two genera by the shape of their carapace – significantly wider than long in *Pachyballus* and elongate in *Peplometus* (Figs 7, 8). However, Simon depicted this feature on *Pachyballus transversus*, which has the widest carapace among its congeners. In most species that can be assigned to this genus the ratio of carapace width and length is approximately 1:1 and it may overlap with the proportions seen in *Peplometus*. A better character allowing the separation of these genera is the shape of abdomen. It is clearly elongated in *Peplometes*, whereas in *Pachyballus* the length of abdomen is equal to its width. The other reliable feature for telling apart the two genera, which may be applied only to males though, is the form of the tibia I. These tibiae in *Pachyballus* are not modified (Fig. 129), but in *Peplometus* are always conspicuously altered. They are strongly thickened and often flattened (Figs 130–133), or considerably elongated, but in this case the metatarsus has a basal process (Figs 134, 135). Tibia I in *Pachyballus* bears dense and long hairs on ventral surface (Fig. 129), whereas in *Peplometus* it has a “brush” of long leaf-like setae (Figs 132–135). The structure of tibia I of males may even be used in distinguishing *Peplometus* species. Identifying females in this genus is very difficult in general and the results may be doubtful. Conversely, species recognition by *Pachyballus* is relatively straightforward in females, where a combination of morphological characters and genitalia conformation is used. Determination of the males is sometimes impossible, because the structure of their palpal organs is extremely similar and there often lack reliable distinguishing morphological features. As a consequence of this, we were able to construct the key only for *Pachyballus* females and males of *Peplometus*. Most probably, many taxonomic intricacies will be possible to solve only using molecular methods. Fresh material for analysis of the two genera will, however, be difficult to obtain.

**Table 1.** Features for distinguishing *Pachyballus* and *Peplometus*.

**Genus**	**shape of the abdomen**	**structure of leg I in male**
* Pachyballus *	rounded or heart-shaped (width-length ratio = ca. 1:1)	tibia not modified, with long setae ventrally
* Peplometus *	clearly elongated (width-length ratio ≤ 0.8)	tibia very robust with dense feather-like setae ventrally, or elongated with same setae and metatarsus with a dorsal hump

## Taxonomic account

### Pachyballus

Taxon classificationAnimaliaAraneaeSalticidae

Simon, 1900

EFE6C941-06F2-5013-A6AD-6CF4AE55626F

Pachyballus Simon 1900: 399; 1901: 486.

#### Type species.

*Pachyballus transversus* Simon, 1900.

#### Diagnosis.

*Pachyballus* is closely related to *Peplometus*. From the latter genus it can easily be separated by the rounded abdomen (elongated in *Peplometus*), by the form of leg I in males (not modified) and by the absence of the leaf-like setae on tibia I.

#### Description.

Small to medium-sized spiders (ca. 3.0–5.0 mm length), with very flat body, covered with tough highly sclerotised integument. Body colouration in the majority of species dark brown or black with metallic lustre, only legs (especially in females) lighter. Carapace rounded, its width usually slightly larger than the length, eye field clearly trapezoid. Posterior part of carapace covered with abdomen. Chelicerae with three (exceptionally two) teeth on promargin and two to four teeth on retromargin, basally fused together. Abdomen short and wide, heart-shaped or rounded, its length to width ratio is 0.8–1.1, clearly wider than carapace, with straight anterior border. Abdominal dorsum totally covered with strongly sclerotised scutum, its edges sloping so that the abdomen has shape of a shallow bowl. Venter with a narrow scutum along anterior margin, its lobes extending laterally as lobes. A second, trapezoid scutum in posterior part, numerous very small sclerotised bumps on both sides of abdomen (Fig. 1). Legs short, first pair slightly bigger in males, with enlarged femora. Tibia I not modified, setae on its ventral side not so dense as in *Peplometus*. Tarsus of the female palp usually black. Male palp with oval bulb, embolus spirally coiled around its tip (Fig. 3). Tibial apophysis thin and straight. Epigyne with large semi-circular depression (atrium), usually divided by epigynal septum, copulatory ducts usually long, forming a spiral in their initial parts and with several complex loops distally.

### Key to *Pachyballus* (females only)

**Table:** 

1	Ventral scutum in posterior half of the abdomen present (Fig. 2)	**2**
–	Ventral scutum in posterior half of the abdomen absent.	***P. variegatus***
2	Ventral posterior scutum trapezoid, large, its width about half of abdomen width	**3**
–	Ventral scutum small, its width not more than third of abdomen width (Fig. 61)	***P. miniscutulus***
3	Carapace clothed in white hairs, epigynal depression wide and short (Fig. 16)	***P. caelestis***
–	White hairs absent from carapace, epigynal depression long	**4**
4	Copulatory ducts relatively short, forming no more than a single loop	**5**
–	Copulatory ducts very long, forming several loose loops	**6**
5	Loop of the copulatory ducts tight (Fig. 28)	***P. castaneus***
–	Loop of the copulatory ducts loose (Fig. 73)	***P. mombasensis***
6	Abdomen with a clear, contrasting pattern (Fig. 81)	***P. ornatus***
–	Abdomen uniformly coloured	**7**
7	Palps dark, abdomen rounded (Fig. 104)	***P. transversus***
–	Palps yellow, abdomen ovoid (Fig. 33)	***P. flavipes***

### Pachyballus caelestis sp. nov.

Taxon classificationAnimaliaAraneaeSalticidae

106CD250-4466-54E3-84E7-0DB5EED3B7B7

http://zoobank.org/DD252B66-8F33-4266-B6A1-670C09BA3D3F

Figures 9–13 , 14–17 , 193

#### Holotype.

Congo D.R. • ♀; Mayombe, Bas Congo, Luki Forest Reserve; 5°37'S, 13°05'E; 23.IX.2007; D. De Bakker and J.P. Michiels leg.; canopy fogging, old secondary rainforest; MRAC 226 102.

#### Paratypes.

Congo D.R. • 1♀; same locality as the holotype; 25.IX.2007; canopy fogging, old secondary rainforest; MRAC 226 107 • 1♀; the same locality; 1.X.2007; canopy fogging, primary rainforest; MRAC 226 114 • 1♂; the same locality; 13.XI.2006; canopy fogging, primary rainforest; MRAC 226 113.

#### Diagnosis.

This species is covered very densely with short hairs, which is the best feature to distinguish it from congeners. Male palpal organ has a characteristic embolus that forms a considerably high and narrow coil similar to that of *P. castaneus*. The coil comprises four loops (two and a half in the latter species). The female is distinctive in having the epigyne with broad, short ridge on posterior edge of the epigynal depression; the copulatory ducts are relatively straight (they do not form any loops).

#### Etymology.

The specific name is from Latin, meaning “soaring” and refers to this species living high in a forest canopy.

#### Description.

**Male.** Measurements. Cephalothorax: length 1.4, width 1.5, height 0.5. Eye field: length 0.7, anterior width 1.2, posterior width 1.5. Abdomen: length 1.8, width 2.0.

**Figures 9–13.**
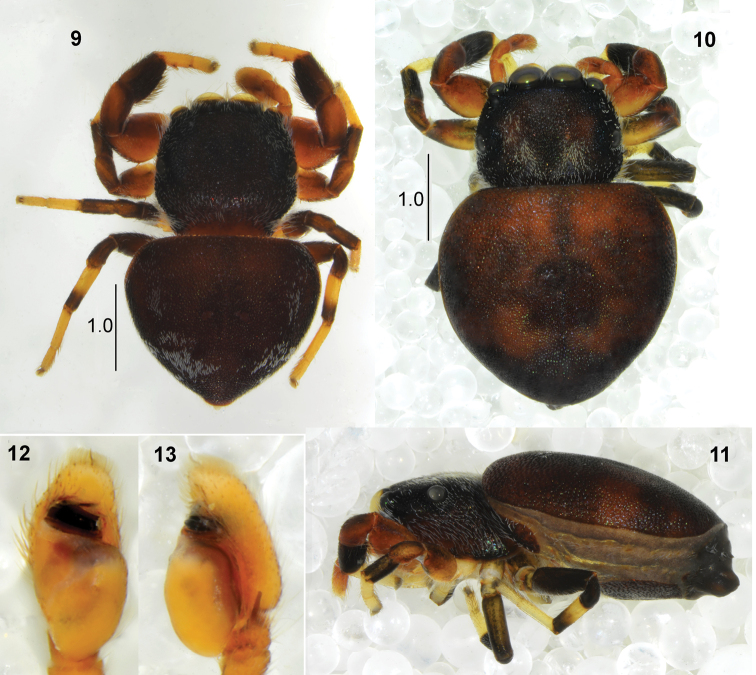
*Pachyballus caelestis* sp. nov. **9** male, habitus, dorsal view **10** female, habitus, dorsal view **11** female, habitus, lateral view **12** palpal organ, ventral view **13** palpal organ, lateral view.

**Figures 14–17.**
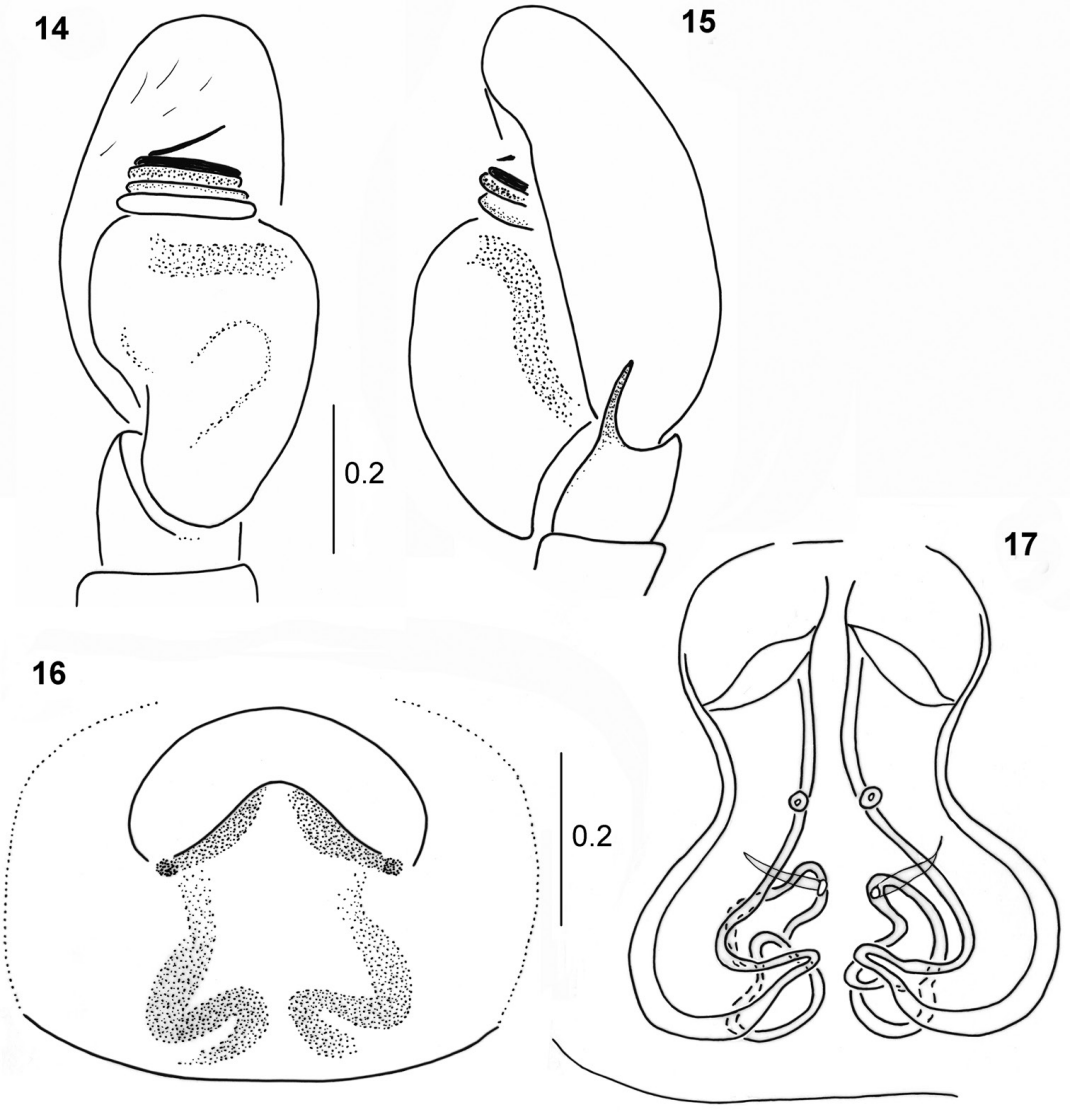
*Pachyballus caelestis* sp. nov. **14** palpal organ, ventral view **15** palpal organ, lateral view **16** epigyne **17** internal structure of epigyne.

General appearance as in Fig. 9. Small, very flat spider with strongly sclerotised, pitted integument. Carapace black, clothed in dense short light hairs. Eye field trapezoid, distance between anterior lateral eyes shorter than between posterior laterals. Eyes encircled by white scales. Clypeus low, with a few white hairs. Chelicera with two teeth on promarginal edge and four retromarginal teeth. Endites and labium light brown with whitish tips. Abdomen heart-shaped, wider than long, blackish with white scales on sides and posterior part. Anterior margin of abdomen covers posterior part of carapace. Venter brown, with typical scuta. Spinnerets black. Legs I the stoutest, femur and patella brown, tibia slightly thickened, black with long dark setae ventrally, metatarsus and tarsus yellowish. Tibia with one short ventral spine distally, metatarsus with two pairs of ventral spines. Legs II the same in colour as the first pair. Legs III and IV brown with yellow distal segments. Pedipalp yellowish, its structure similar to that in other species, embolus forms a considerably high and narrow coil that comprises four loops as in Figs 12–15.

**Female.** Measurements. Cephalothorax: length 1.1–1.2, width 1.3–1.4, height 0.5. Eye field: length 0.6–0.7, anterior width 1.0–1.1, posterior width 1.2–1.3. Abdomen: length 2.0–2.2, width 2.2–2.4.

General appearance as in Figs 10, 11. Slightly larger than male, shape of body similar. Carapace covered with white hairs. Colouration of abdomen a little lighter than in male, brown, blackish in the mid part and along edge. Palps light brown. Epigyne oval, central depression divided posteriorly by short, wide ridge (Fig. 16). Copulatory ducts wide, spermathecae slightly smaller than in other species, small accessory glands opening into copulatory ducts (Fig. 17).

#### Distribution.

Known only from the type locality (Fig. 193).

#### Remarks.

All specimens were collected by fogging. Probably, this species lives high in the forest canopy.

### Pachyballus castaneus

Taxon classificationAnimaliaAraneaeSalticidae

Simon, 1900

EECBB1E0-78F3-5A29-9F50-9DD3AEBCADF1

Figures 6 , 18–21 , 22–28 , 129 , 193

Pachyballus castaneus Simon 1900: 400 (♀).

#### Neotype.

South Africa • ♀; KwaZulu-Natal, Ulundi, Ophathe Game Reserve; 28°23'S, 31°24'E; 3.X.2008; C. Haddad leg.; overgrazed savanna, beating shrubs; NCA 2008/4147.

#### Paraneotypes.

South Africa • 1♀; together with neotype • 1♂ 1♀; the same locality as neotype; 3.X.2008; C. Haddad leg.; overgrazed savanna, beating, shrubs; NCA 2008/4140.

**Figures 18–21.**
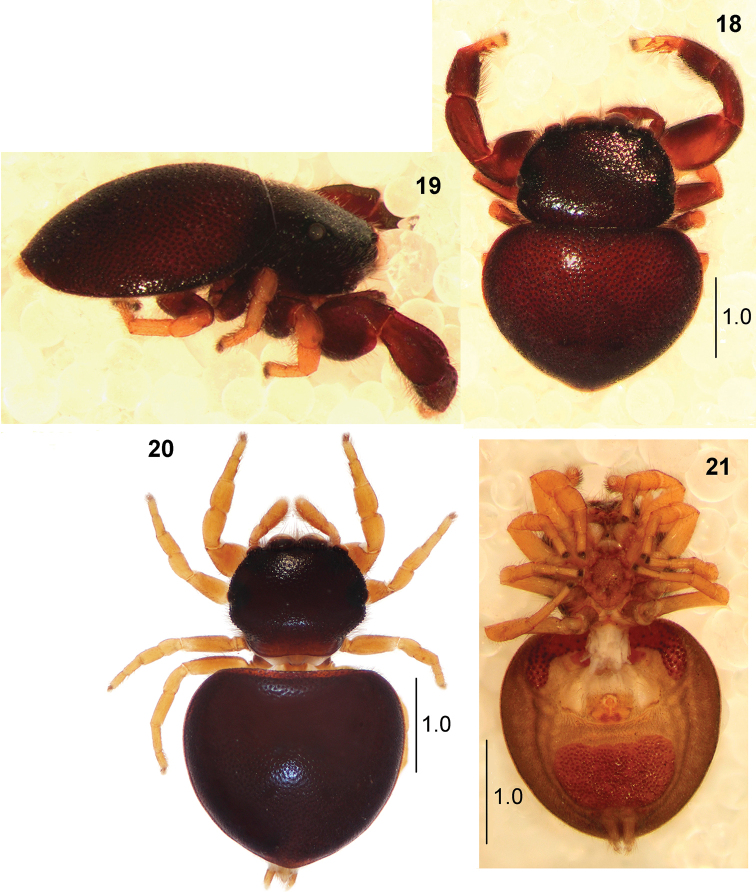
*Pachyballus castaneus***18** male (specimen from South Africa), habitus, dorsal view **19** male habitus, lateral view **20** female, habitus, dorsal view **21** female, neotype, habitus, ventral view.

#### Other material examined.

South Africa • 1♂; the same locality as neotype; 2.X.2008; NCA 2008/4167 • 1♂ 2♀ 8 imm.; the same locality; 500 m a.s.l.; rocky mountainside; NCA 2008/4154 • 3♂ 6 imm.; the same locality; 1.X.2008; NCA 2008/3993 • 1♂ 3 imm.; the same data; NCA 2008/3971 • 1♂; KwaZulu-Natal, iSimangaliso Wetland Park, Crocodile Centre; 28°21'S, 32°25'E; 14.V.2012; J.A. Neetling and C. Luwes leg.; canopy fogging, wetland, *Breonadia salicina*; NCA 2012/5736 • 2♀; the same locality, St. Lucia; 28°23'S, 32°25'E; 13.V.2012; J.A. Neetling and C. Luwes leg.; canopy fogging, coastal forest, *Trichilia dregeana*; NCA 2012/4019 and NCA 2012/5737 • 1♀; Lake St. Lucia, Fanies Island; 28°06'S, 32°27'E; 1.VII.1993; F.J. van der Lingen leg.; 200 m from lake campsite, W shore, in dense bush thicket; NMBA 08069 • 1♀; Mkuze, Banghoek Lodge; 27°45'S, 32°08'E; 17.V.2012; J.A. Neetling and C. Luwes leg.; canopy fogging, Bushveld, *Acacia karroo*; NCA 2012/3965 • 1♂; Ndumo Game Reserve; 26°55'S, 32°19'E; 30.VI.2009; R. Lyle leg.; sand forest, beating foliage; NCA 2009/3660 • 1♀; Sihangwane, Tembe Elephant Park, 40 km from Kosi Bay; 27°02'S, 32°25'E; 18.XI.1988, R. Harris leg.; NCA 94/828 • 1♀; the same locality; 6.II.2008; R. Lyle and R. Fourie leg.; beating, afromontane forest; NCA 2008/505 • 1♂ 1♀; Hellsgate; 28°00'S, 32°48'E; 23.VIII.2004; J. Esterhuizen leg.; NCA 2010/155 and NCA 2010/156 • 1♀; Mpumalanga Prov., Nelspruit, Agricultural College; 25°27'S, 30°59'E; 12.XI.1999; P. Stephen leg.; beating, citrus; NCA 2000/223 • 1♀; Wildlife College, 10 km from Orpen Gate of Kruger National Park; 24°28'S, 31°23'E; 11.X.2000; W. Breytenbach leg.; beating *Euclea divinorum*; NCA 2003/626 • 1♂; Limpopo Prov., Little Leigh; 22°56'S, 29°22'E; 22.XI.2005; B. van der Waal leg.; branch beating, gallery forest; NCA 2009/2232. Zimbabwe • 3♂; Mashonaland; Workman coll.; MCZ • 1♂; Harare; 17°50'S, 31°10'E; 2.III.2012; M. Cumming leg.; suburban garden, dropped from tree; NMZ.

#### Diagnosis.

The male is indistinguishable from the males of *P. flavipes* and *P. mombasensis* by body shape and colouration, but its bulb is slightly narrower than in these species and the embolic spiral is tightly convoluted; width of the basal embolic loop equals only a half of tegulum width, whereas in both other species it is as wide as tegulum. The female can be separated from congeners in having copulatory ducts compactly arranged and not forming loose loops (see Fig. 28).

**Figures 22–28.**
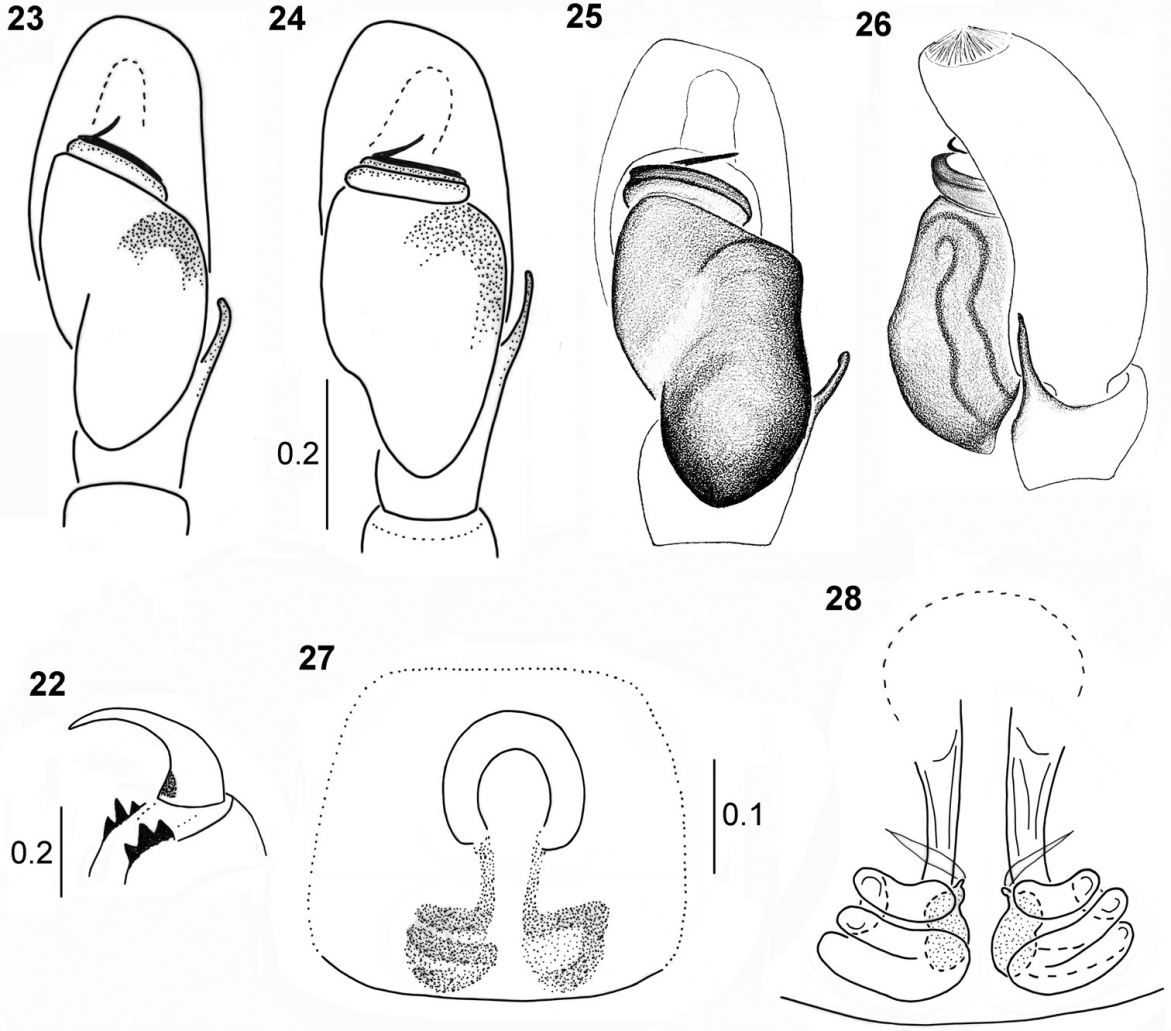
*Pachyballus castaneus***22** cheliceral dentition **23–25** palpal organ, ventral view (24 specimen from Zimbabwe) **26** palpal organ, lateral view **27** epigyne **28** internal structure of epigyne.

#### Redescription.

**Male.** Measurements. Cephalothorax: length 0.9–1.3, width 1.3–1.9, height 0.5–0.6. Eye field: length 0.6–0.7, anterior width 1.1–1.4, posterior width 1.3–1.9. Abdomen: length 1.7–2.2, width 1.8–2.6.

General appearance as in Figs 18, 19. Small spider with flattened body covered with strongly sclerotised and pitted integument (Fig. 6). Carapace dark brown, eye field on more or less half of carapace, vicinity of eyes black. A few long bristles at anterior eyes. Posterior edge of carapace covered by abdomen. Chelicerae with three teeth on both margins (Fig. 22). Mouth parts brown, sternum oval, brown, clypeus extremely low. Abdomen dark brown, heart-shaped, as wide as long, dorsal scutum turned back via margins to venter (Fig. 19), ventral scuta typical; the anterior one narrow with lateral extensions, posterior trapezoid. Numerous small bumps on sides. Colouration dark brown to black, iridescent, integument clearly pitted, short hairs on edges of carapace, dense bristles near eyes. Spinnerets brown. Legs I the thickest (Fig. 19), brown (except yellow tarsi and metatarsi), femur, patella and tibia slightly thickened, tibia slightly flattened dorsally, covered with dense setae ventrally. Tibiae and metatarsi with two pairs of short stout spines ventrally. Other legs yellow, with brownish femora. Pedipalp brown, its structure as in Figs 23–26, slightly narrower than in congeners. Palpal tibia short with single thin straight apophysis, bulb oval, embolus thin, long, spirally coiled on bulb tip.

**Female.** Cephalothorax: length 0.9–1.4, width 1.2–1.4, height 0.5–0.6. Eye field: length 0.5–0.7, anterior width 0.9–1.1, posterior width 1.2–1.4. Abdomen: length 1.7–2.0, width 1.8–2.1.

General appearance as in Figs 20, 21. Similar to male, abdomen almost round or oval, but its anterior margin almost straight. Abdominal venter with two scuta as in male (Fig. 21). All legs and palps dark yellow. Epigyne typical, with horseshoe-shaped anterior depression (Fig. 27). Internal structures relatively simple, copulatory ducts shorter than in congeners, compactly arranged, spermathecae strongly sclerotised (Fig. 28).

**Immature specimens.** Similar to adults, abdomen covered dorsally with one large scutum.

#### Distribution.

Known from South Africa and Zimbabwe (Fig. 193).

#### Designation of neotype.

*Pachyballus castaneus* was originally described from Natal (South Africa) on the basis of a single female. The type specimen was lost (the collection manager informed us that the type could not be found in Simon’s collection in MNHN). The original description is very superficial (only body size and colouration of legs are mentioned) and insufficient for identification of the species. Colouration of legs varies within different *Pachyballus* species (see Figs 32, 33, 104–106), so it cannot be used as a taxonomic character. The neotype, a female that originates from the same province as the type, is herein designated to stabilise the nomenclature.

#### Remarks.

The male of this species is described here for the first time.

### Pachyballus flavipes

Taxon classificationAnimaliaAraneaeSalticidae

Simon, 1910

2E6F478F-E15E-5F77-82F3-8FE072858462

Figures 1 , 3 , 5 , 29–35 , 36–40 , 41–46 , 47–50 , 51–57 , 194

Pachyballus flavipes Simon 1910: 414 (♀); Lessert 1925: 434, f. 7–9 (♀); Wanless and Clark 1975: 290, f. 27–28 (♀); Dawidowicz and Wesołowska 2016: 449.
P. flavipes aurantius Caporiacco 1949: 464 (♀), syn. nov.
Pachyballus cordiforme Berland and Millot 1941: 396, f. 90 (♂), syn. nov.
Pachyballus cordiformis Wesołowska and Cumming 2011: 87, f. 40–45 (♂).

#### Neotype.

Cameroon • ♀; Biniiba, Bétaré-Oya; 5°36'N, 14°05'E; 20.VII.1949; B. Malkin leg.; CAS.

#### Other material examined.

Angola • 1♀; Hulla prov., Caconda; 13°46'S, 15°05'E; 30.IX.1949; B. Malkin leg. CAS. Botswana • 1♀; Okavango Delta, Pom-Pom Camp; 19°18'S, 22°54'E; VII.2001; E. Kassimatis leg.; sweeping; NCA 2009/5688. Cameroon • 1♀; Faro Game Reserve; 8°30'N, 12°30'E; 25.IV.2007; R. Jocqué, K. Loosveldt, L. Baert and M. Alderweireldt leg.; gallery forest, sieving; MRAC 221 442. Congo D.R. • 1♀; South Kivu prov., Itombwe; 3°15'S, 28°50'E; 3200 m a.s.l.; XII.1958; N. Leleup leg.; forest with *Hagenia* and bamboo; MRAC 113 229 • 1♀; Semliki river valley; 9.VIII.1968; R.P.M. Lejeune leg.; MRAC 135 557 • 2♂; Mayombe, Bas Congo, Luki Forest Reserve; 5°37'S, 13°05'E; 17.IX.2007; D. De Bakker and J.P. Michiels leg; old secondary rainforest, fogging; MRAC 226 092 • 1♂; the same data; 18.IX.2007; MRAC 226 097 • 1♂; the same data; 20.IX.2007; MRAC 226 095 • 1♀; the same data; 23.IX.2007; MRAC 226 103 • 2♀; the same data; 18.IX.2007; MRAC 226 096 • 1♀; the same data; 20.IX.2007; MRAC 226 094 • 2♀; the same data; 21.IX.2007; MRAC 226 093 • 1♂ 2♀ 2 subad. ♂; the same data; 17.IX.2007; MRAC 226 101 • 2♂; the same locality; 28.IX.2007; primary rainforest, fogging; MRAC 226 109 • 1♀; the same data; 1.X.2007; MRAC 226 116 • 1♂ 1♀; the same data; 1.X.2007; MRAC 226 120 • 3♀; the same data; 7.XI.2006; MRAC 220 945 • 3♀; the same data; 13.XI.2006; MRAC 220 971 • 1♀; the same locality; 14.IX.2007; secondary rainforest, beating; MRAC 223 432 • 2♂; Kivu prov., Ngoma; 4°24'S, 26°05'E; L. Burgeon leg.; MRAC 15 552/15 553 • 1♀; Kivu prov., Rutshuru, Kako; 1°11'S, 29°27'E; IX.1932; L. Burgeon leg.; MRAC 31 267 • 1♀; Kivu prov., Sanga plateau; 4°50'S 14°58'E; N. Leleup leg.; MRAC 119 189. Gabon • 1♂; Biso-Binam [Biso stream?]; 0°52'N, 11°39'E; 3.XI.1985; A. Pauly leg.; on *Borreria verticillata*; MRAC 172 765. Guinea • 1♂; Nimba Mts, “Mare d’hivernage” (in UNESCO Biosphere reserve); 7°38'N, 8°27'W; 1650 m a.s.l.; 1.X.2008; D. van den Spiegel leg.; wet grassland with dispersal shrubs; MRAC 225 978. Ivory Coast • 1♂; Man; 7°24'N, 7°33'W; VIII,1937; J. Millot leg.; type of *P.*cordiformis: only two, left palps and legs left; MNHN • 1♀; Bingerville; 5°21'N, 3°54'W; VIII.1962; J. Decelle leg.; MRAC 122.004. Kenya • 1♂; Kwale prov., 30 km S to Mombasa; 4°10'S, 39°40'E; 12.XI.1992; V. Roth leg.; CAS • 1♀; Kitale; 1°01'N, 35°00'E; 2000 m a.s.l.; 23.I.1938; MEU • 1♀; Nairobi; 1°17'S, 36°49'E; 12.I.1970; on bamboo; SMF • 1♂; Mt Elgon, E slope; 1°07'N, 34°31'E; 2130 m a.s.l.; 6.I.1938; NHRS • 1♀; Mt Elgon, Salt lake estate; 2100 m a.s.l.; 17.XII.1937; *Acacia* steppe; MEU. Tanzania • 1♀; Kilimanjaro, Kibonoto; 3°11'S, 37°06'E; Sjöstedt leg.; NHRS. Uganda • 1♀; Victoria Lake, Gaya Bay, Island Buvuma; 0°13'N, 33°16'E; III.1968; E. Vertriest leg.; MRAC 134 737. South Africa • 1♂; Free State, Weltevreden Nature Reserve; 28°57'S, 26°23'E; 15.VIII.2006; H. Kilion leg.; NCA 2007/3429 • 1♀; Mpumalanga Prov., Nelspruit, Agricultural College; 25°27'S, 30°59'E; 22.XII.1998; P. Stephen leg.; on grapefruit; NCA 99/160 • 1♀; Limpopo Prov., Nylsvley Nature Reserve; 24°39'S, 28°41'E; 7.III.1998; sweeping, grass; A.S. Dippenaar leg.; NCA 98/586. Zambia • 1♂; 30km SW of Mkushi; 13°43'S, 29°15'E; 1390 m a.s.l.; 22.IX.2009; J.Lenz leg.; NMZ. Zimbabwe • 1♂ 2 imm.; Batoka Gore; 17°57'S, 26°04'E; 30–31.X.1990; V. Roth leg.; CAS • 1♂ 2♀; Matabeleland, Doddieburn Dam; 21°24'S, 29°21'E; 12.XII.1985; J. Minshull leg.; NMZ A4202 • 5♀ 2 subad. ♂ 8 imm.; Matetsi safari Area, Tshowe river rapids; 18°31'S, 25°52'E; 5.XII.1988; J. Minshull leg.; NMZ A6873 • 2♂; Matetsi safari Area, Kasetsheti Weirs; 18°01'S, 25°49'E; 11.X.1988; F. Nyati leg.; NMZ A6759.

**Figures 29–35.**
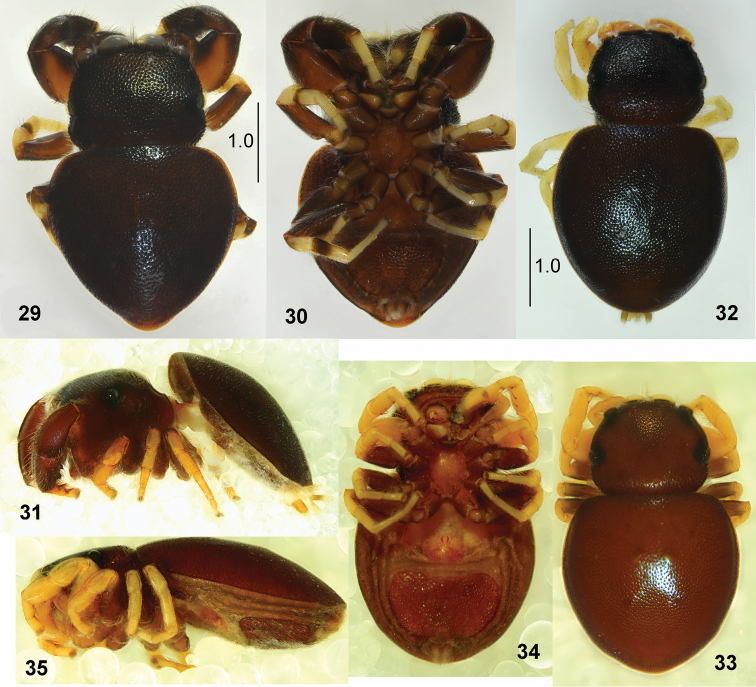
*Pachyballus flavipes***29** male, habitus, dorsal view **30** male, habitus, ventral view **31** male, habitus, lateral view **32, 33** female, habitus, dorsal view **34** female, habitus, ventral view **35** female, habitus, lateral view (**29, 30, 32** specimen from Congo **31** specimen from Kenya **33–35** specimen from Cameroon).

#### Diagnosis.

The male is almost indistinguishable from that of *P. mombasensis*, though it differs a little by having a protruding tibial apophysis while in the latter species tibial apophysis is adpressed to cymbium. The female resembles females of *P. castaneus* and *P. mombasensis*, but has very long copulatory ducts, forming several loops, whereas in two other species these ducts are relatively short (cf. Fig. 51 with Fig. 73 and Fig. 28).

#### Redescription.

**Male.** Measurements. Cephalothorax: length 1.2–1.6, width 1.4–1.7, height 0.5–0.6. Eye field: length 0.6–0.8, anterior width 1.1–1.4, posterior width 1.4–1.7. Abdomen: length 1.9–2.5, width 1.8–2.3.

**Figures 36–40.**
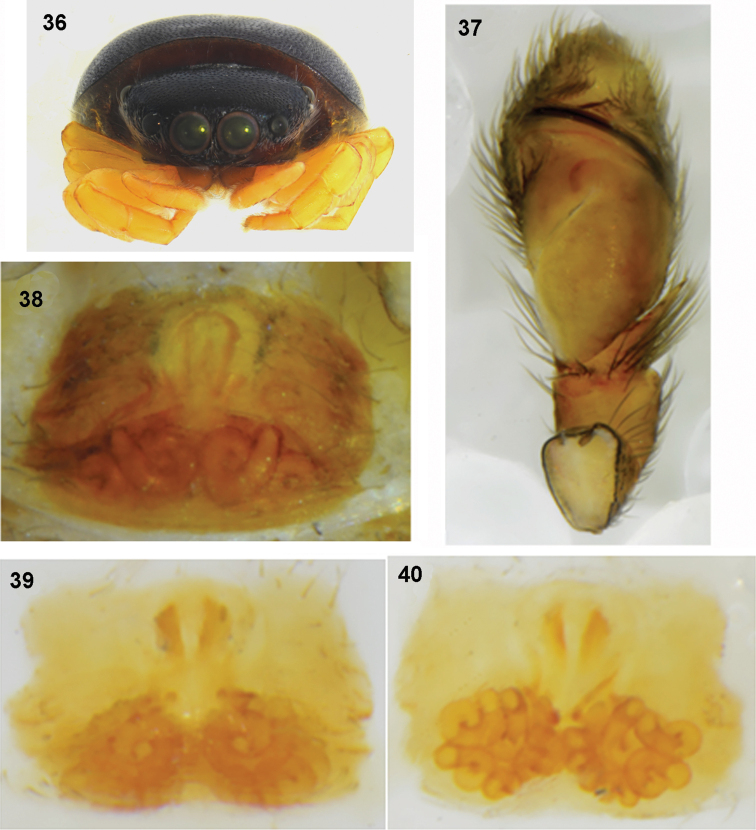
*Pachyballus flavipes* (specimen from Congo) **36** female, habitus, frontal view **37** palpal organ, ventral view **38** epigyne **39** internal structure of epigyne, ventral view **40** internal structure of epigyne, dorsal view.

**Figures 41–46.**
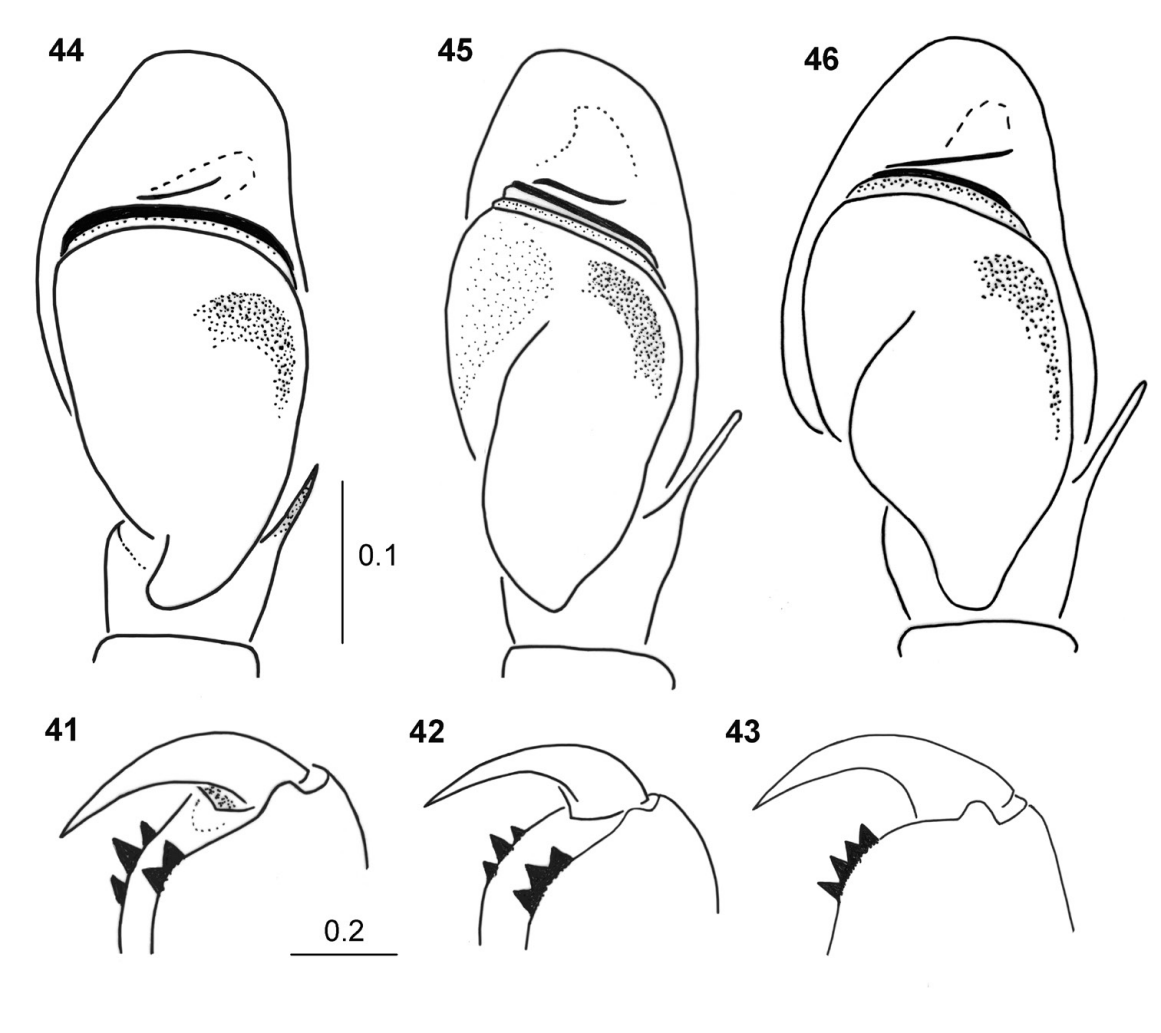
*Pachyballus flavipes*, male **41–43** variation of cheliceral dentition **44–46** palpal organ, ventral view (**44** specimens from Congo **45** specimen from Gabon **46** type of *P. cordiformis*).

General appearance as in Figs 29–31. Shape of body typical for *Pachyballus*; small, flat, with heart-shaped abdomen. Body covered with strongly sclerotised and clearly pitted integument. Colouration of body brown to black dorsally and dark brown ventrally, dorsum iridescent, almost hairless. Some light hairs near anterior eyes and below anterior median eyes. Anterior margin of abdomen covers distal part of carapace. Venter with typical scuta and small sclerotised bumps (Fig. 1). Cheliceral dentition variable, with three teeth on promargin and two, three or (exceptionally) four on retromargin (Figs 41–43). Tips of mouth parts whitish. First pair of legs the stoutest, femora and patellae dark brown, tibiae black with dense long dark setae ventrally and two pairs of very short thick spines (Fig. 31), dorsal part of tibia slightly flattened, metatarsi and tarsi creamy. Other legs with clear contrasts: brown femora, other segments light, only sometimes dark ring on tibiae proximally (Fig. 30). Palps dark, tip of cymbium light. Structure of palpal organ as in Figs 3, 37, 44–46.

**Female.** Measurements. Cephalothorax: length 1.2–1.3, width 1.3–1.4, height 0.5. Eye field: length 0.6–0.8, anterior width 1.1–1.3, posterior width 1.3–1.4. Abdomen: length 1.9–3.1, width 1.7–2.9.

General appearance as in Figs 32–36. Similar to male, abdomen more oval. Anterior median eyes surrounded by short light hairs. All legs yellowish (Fig. 35), only femora brownish (or yellow with darker streak). Palps yellow (Figs 32, 36). Epigyne as in Figs 5, 38, 47–50, with horseshoe-shaped anterior depression. Internal structure of epigyne as in Figs 39, 40, 51–57, inlet part of copulatory ducts wide.

**Immature specimens.** Similar to adults, abdomen dorsally covered with one large scutum.

#### Distribution.

Species widely distributed in Africa (Fig. 194). Some of the records from Kenya were already mentioned in Dawidowicz and Wesołowska (2016).

#### Synonymisation.

Caporiacco (1949) described *P. flavipes aurantius* from Kenya on the basis of a single female. According to him, the epigyne of this subspecies was the same as in *P. flavipes* and the only difference consisted of colouration. However, this feature is variable. Moreover, according to Berdondini and Whitman (2003) type specimen of *P. flavipes aurantius* kept in the Natural History Museum of Florence collection is juvenile. Thus, we recognise *P. f. aurantius* as a synonym of the name *P. flavipes*.

Type of *P. cordiformis* was destroyed and only two palps and three legs persisted in the vial. Although Berland and Millot (1941) reported that they had collected only a single male by the city Man, the two palps in the sample are left palps, so they must have been taken from two males. Structure of palpal organ and the figure in Berland and Millot (1941) suggest that this species is identical with *P. flavipes*. Therefore, we recognise the name *P. cordiformis* as the synonym of *P. flavipes*.

**Figures 47–50.**
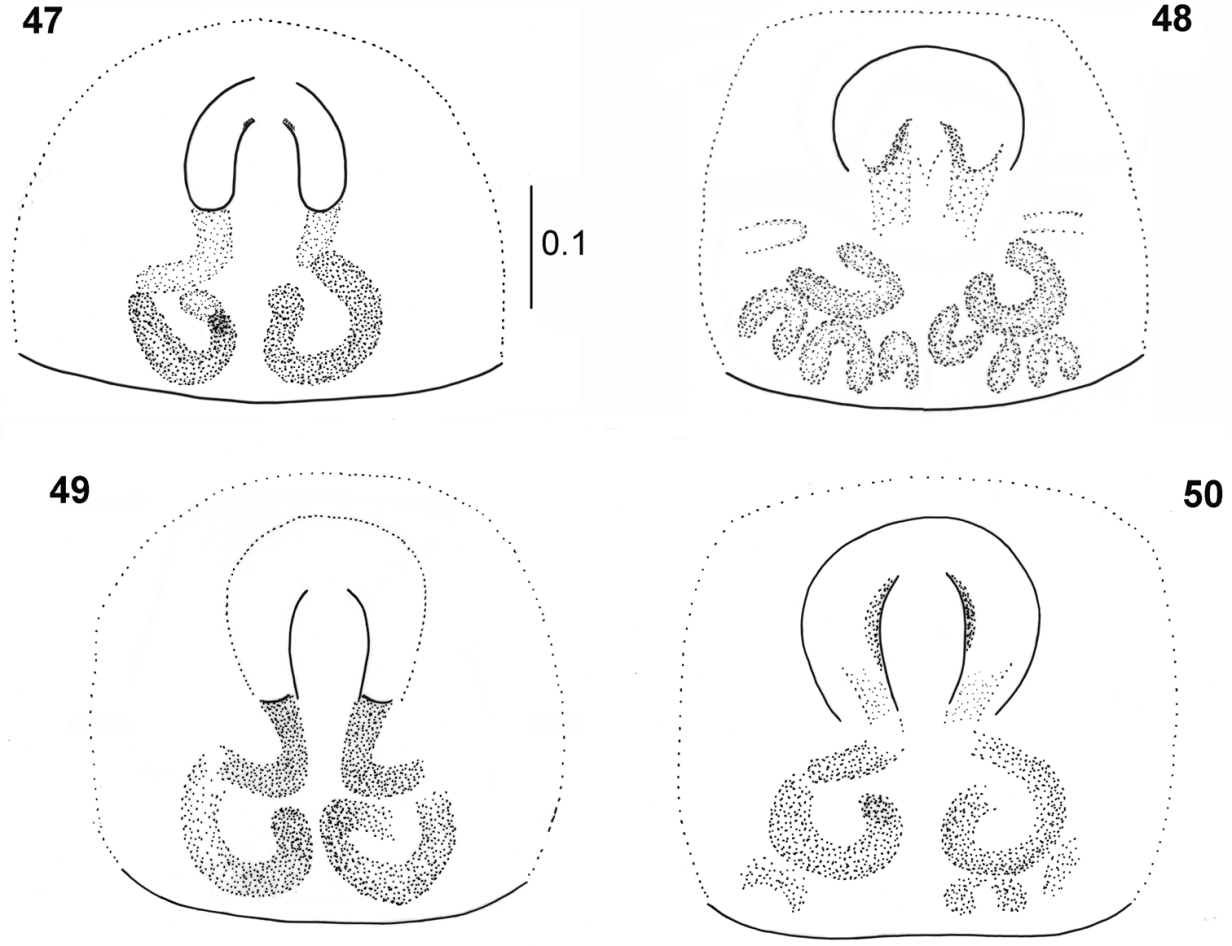
*Pachyballus flavipes*, female, epigyne (**47, 48** specimens from Congo **49** specimen from Kilimanjaro **50** specimen from Cameroon).

#### Designation of neotype.

Simon (1910) described this species from Bioko (Fernando Po) on the basis of a single female. Wanless and Clark (1975) compared a female from Ivory Coast with the type and concluded their conspecificity. Unfortunately, the type specimen was lost (we were informed that the type could not be found in Simon’s collection in MNHN) and the female collected by the latter authors lacked epigyne. Original description was insufficient to recognise the species; only the shape of body and colouration of legs were given. Taking this under consideration it is justified to propose the neotype to stabilise the nomenclature. The neotype, a female collected in Cameroon, the nearest continental country to Bioko (ca. 40 km), is herein designated.

#### Remarks.

This species probably lives both in the forest canopy and in the understory. Given this fact, i.e. the variety of preferred microhabitats, the large geographical range and high variation of cheliceral dentition, it is possible that it consists of several cryptic species. The sole morphology may be insufficient to solve this taxonomic problem and there is a need to support further analysis with molecular methods.

**Figures 51–57.**
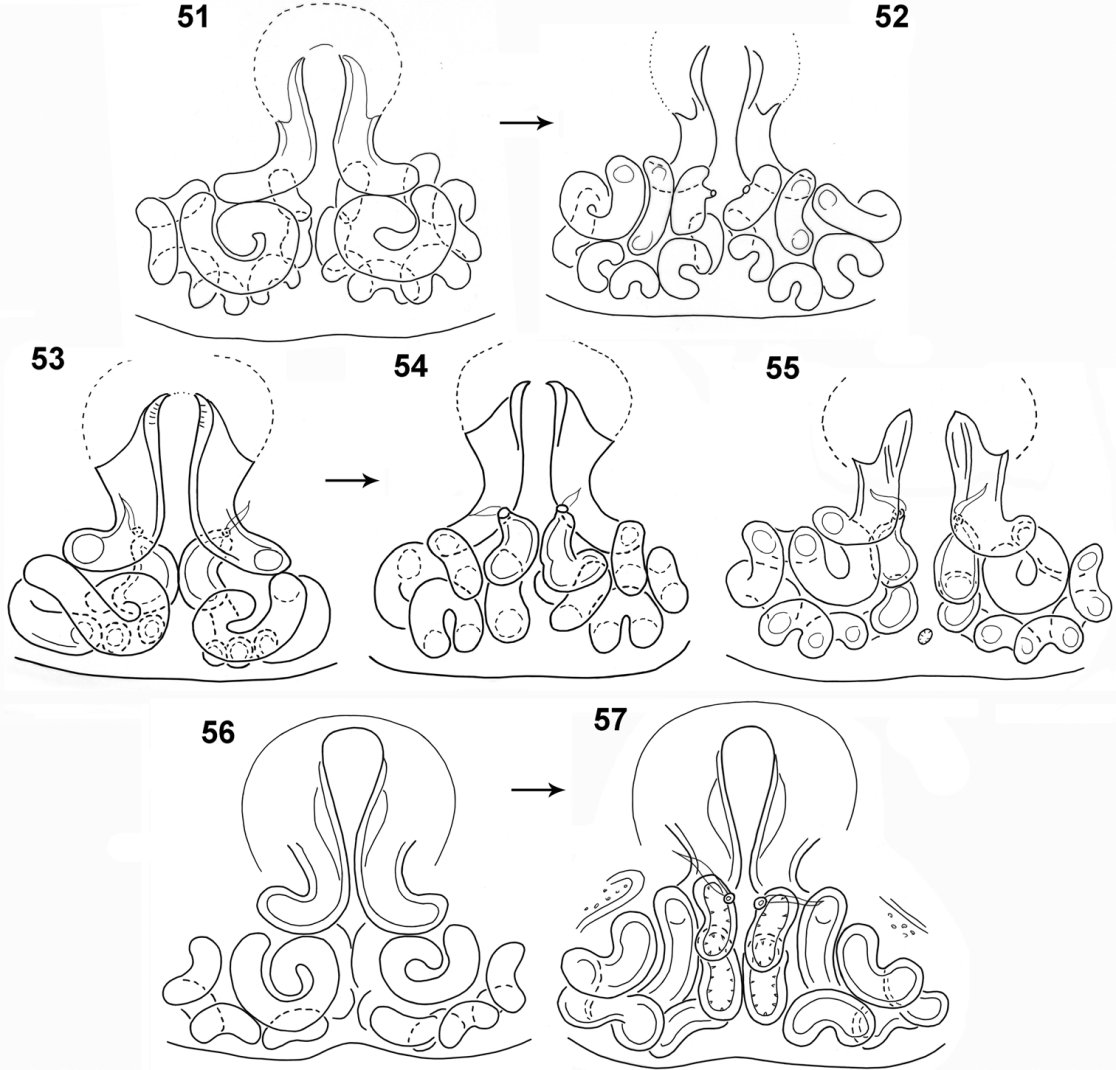
*Pachyballus flavipes*, female, internal structure of epigyne **51, 53, 55, 56** ventral view **52, 54, 57** dorsal view (**51, 52** specimens from Cameroon **53–55** specimen from Congo **56, 57** specimen from Zimbabwe).

### Pachyballus gambeyi

Taxon classificationAnimaliaAraneaeSalticidae

(Simon, 1880)

641B433B-67CC-546F-B0B7-EDFD33433B9F

Homalattus gambeyi Simon 1880: 166 (♂).
Pachyballus gambeyi Simon 1901: 485; Prószyński 1987: fig. on p. 73 (♂).

#### Holotype.

New Caledonia • 1♂; [leg.] Bougier; MNHN 3646; not examined.

#### Remarks.

The holotype was examined by Prószyński (1987). His drawings show that this specimen has palpal organ typical for *Pachyballus*, but morphological characters do not allow any specific identification. Simon described this species from Canala (21°32'S, 165°57'E), central New Caledonia. All species of *Pachyballus* are restricted to Africa, which is thousands kilometers from New Caledonia. This distribution pattern is highly improbable, and the situation might have resulted simply by mislabelling the sample, which is not an uncommon problem in collections. As the provenience of the specimen is doubtful we conclude that the status of *P. gambeyi* remains unclear.

### Pachyballus miniscutulus sp. nov.

Taxon classificationAnimaliaAraneaeSalticidae

CCEEB0F2-4176-5697-82B4-58BC59EC9F13

http://zoobank.org/3DC50D0E-AC8C-4810-827C-CD04B0674B80

Figures 58–61 , 62–67 , 195

#### Holotype.

South Africa • ♂; Free State, Bloemfontein, National Botanical Gardens; 29°02'S, 26°12'E; 12.X.2012; C. Haddad leg.; sweeping, vegetation along stream; NCA 2019/1444.

#### Paratypes.

South Africa • 3♀; together with holotype • 1♀; Free State, Bloemfontein, National Botanical Gardens; VII.2012; L. de Jager and J. van der Merwe leg.; karree litter (*Searsia lancea*), streamside; NCA 2019/1446 • 1♀; the same locality; 19.XII.2012; C. Haddad grassland leg.; pitfall traps; NCA 2013/1635 • 2♀; the same locality; 19.XI.2012; C. Haddad leg.; sweeping, open grassland; NCA 2013/1604 • 4♀; the same locality; 12.X.2012; C. Haddad leg.; sweeping, vegetation along stream; NCA 2012/5707 • 2♀; KwaZulu-Natal, Ithala Game Reserve, picnic site; 27°33'S, 31°19'E; 29.I.2014; C. Haddad leg.; base of grass tussocks; NCA 2013/5098.

**Figures 58–61.**
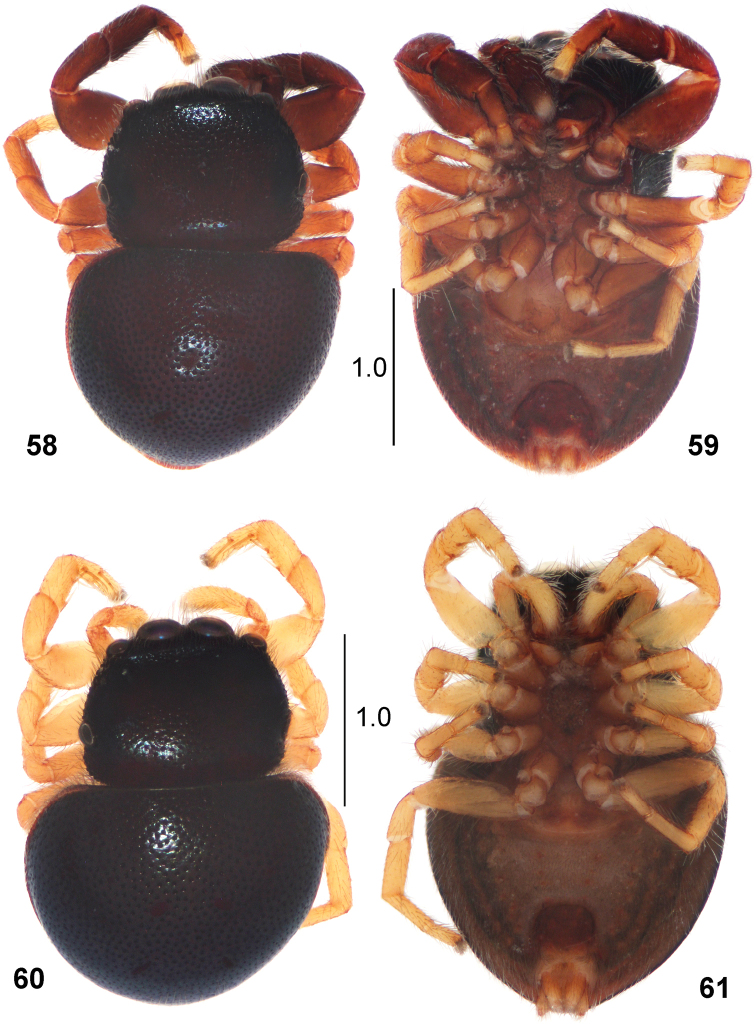
*Pachyballus miniscutulus* sp. nov. **58** male, holotype, habitus, dorsal view **59** male, habitus, ventral view **60** female, habitus, dorsal view **61** female, habitus, ventral view.

#### Diagnosis.

This species is distinctive in having a unique size of ventral posterior scutum (Figs 59, 61) that is clearly smaller than in other species. Its width is equal to spinnerets area (2–3 times larger in the congeners). The female has the epigyne similar to that in *Pachyballus mombasensis*, but the copulatory ducts are longer (cf. Fig. 74 with Figs 66, 67).

#### Etymology.

The specific name is derived from the Latin words “mini-” and “scutum”, meaning “small” and “shield” correspondingly, and refers to the small size of ventral posterior scutum.

#### Description.

**Male.** Measurements. Cephalothorax: length 1.3, width 1.25, height 0.6. Eye field: length 0.7, anterior width 1.0, posterior width 1.2. Abdomen: length 1.7, width 1.7.

**Figures 62–67.**
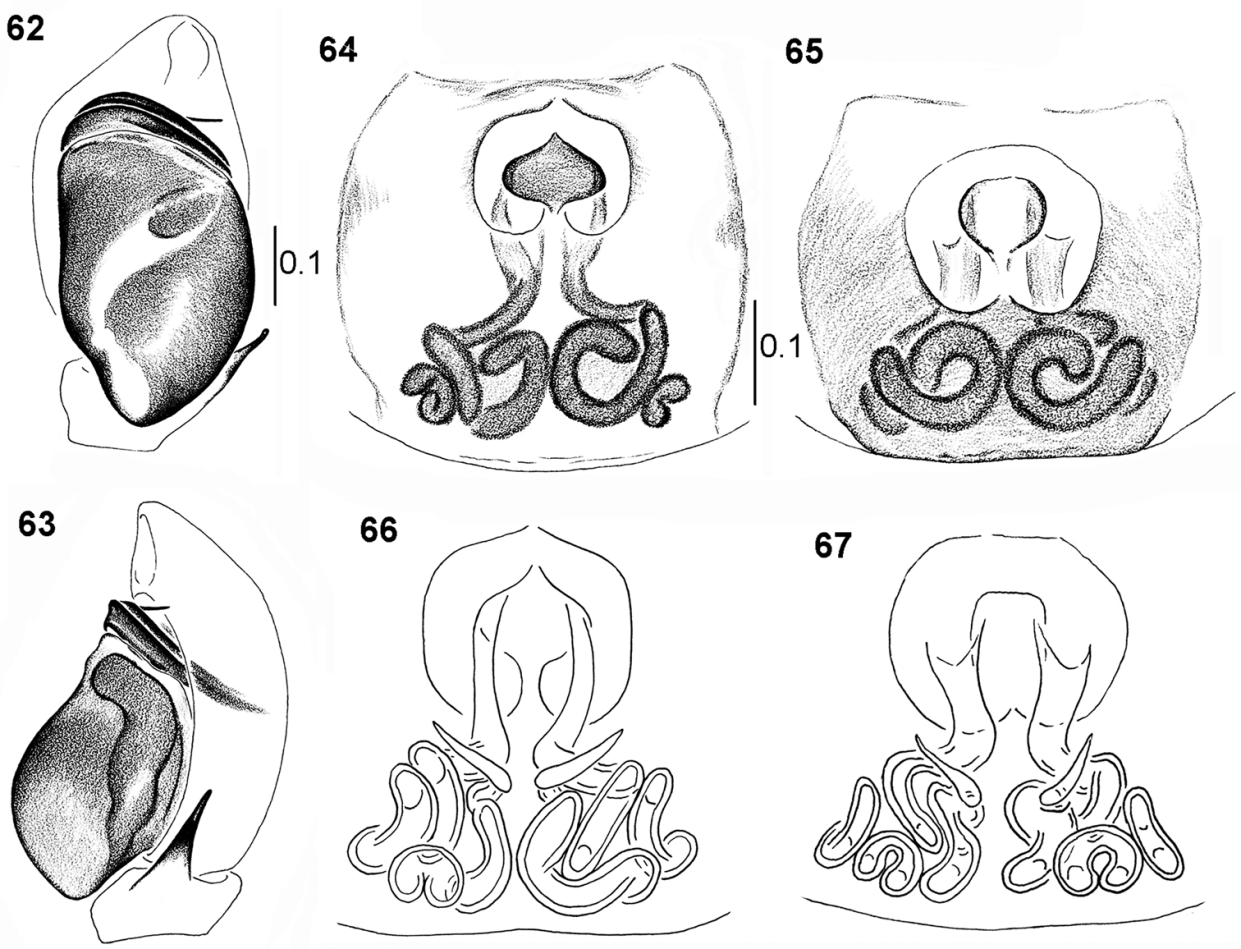
*Pachyballus miniscutulus* sp. nov. **62** holotype, palpal organ, ventral view **63** palpal organ, lateral view **64, 65** epigyne **66, 67** internal structure of epigyne, dorsal view.

General appearance as in Figs 58, 59. Colouration of carapace dark brown, with black rings around eyes, some long bristles at first row of eyes. Chelicerae dark brown. Clypeus and cheeks dark brown, covered with sparse white hairs. Labium and endites yellowish brown, paler apically. Sternum yellowish brown. Abdomen heart-shaped, dark brown dorsally. Venter brownish grey, with a small posterior scutum, ranging at one fifth of abdomen length (Fig. 59). Book-lung covers yellow. Spinnerets yellowish brown. First pair of legs brown with yellow tarsi. Legs II-IV light brown. Leg hairs brown. Structure of palpal organ as in Figs 62, 63, embolic coil wide, comprises 2.5 loops, palpal tibia with protruding apophysis.

**Female.** Measurements. Cephalothorax: length 1.0–1.1, width 1.1–1.2, height 0.5–0.6. Eye field: length 0.5–0.6, anterior width 0.9–1.0, posterior width 1.1–1.2. Abdomen: length 1.8–1.9, width 1.5–1.8.

General appearance as in Figs 60, 61. Similar to male. Posterior ventral scutum small, as in male. All legs and palps yellow. Epigyne as in Figs 64, 65, with spade-like or round central part in semi-circular depression. Internal structure of epigyne as in Figs 66, 67.

#### Distribution.

Known from South Africa only (Fig. 195).

### Pachyballus mombasensis sp. nov.

Taxon classificationAnimaliaAraneaeSalticidae

F48A7FFF-3552-587B-8347-1A49CCA199A9

http://zoobank.org/1A041DD3-803F-49E4-AA4B-F279265DC748

Figures 68–74 , 195

#### Holotype.

Kenya • ♀; Diani Beach, 30 km S to Mombasa; 4°19'S, 39°34'E; 5–19.III.1970; T. Palm leg.; MEU.

#### Paratype.

Kenya • 1♂; together with the holotype; +3 imm.

#### Diagnosis.

The male is indistinguishable from the males of *P. flavipes* and *P. castaneus* by body colouration; it differs from *P. castaneus* in having a wider bulb and wide and low embolic coil (cf. Fig. 70 with Fig. 25); from *P. flavipes* it can be distinguished by the palpal tibial apophysis adpressed to cymbium, (protruding in *P. flavipes*) .The latter character requires verification though, as only single male of *P. mombasensis* is known. The female has relatively short copulatory ducts, forming only a single loop (Fig. 73).

**Figures 68–74.**
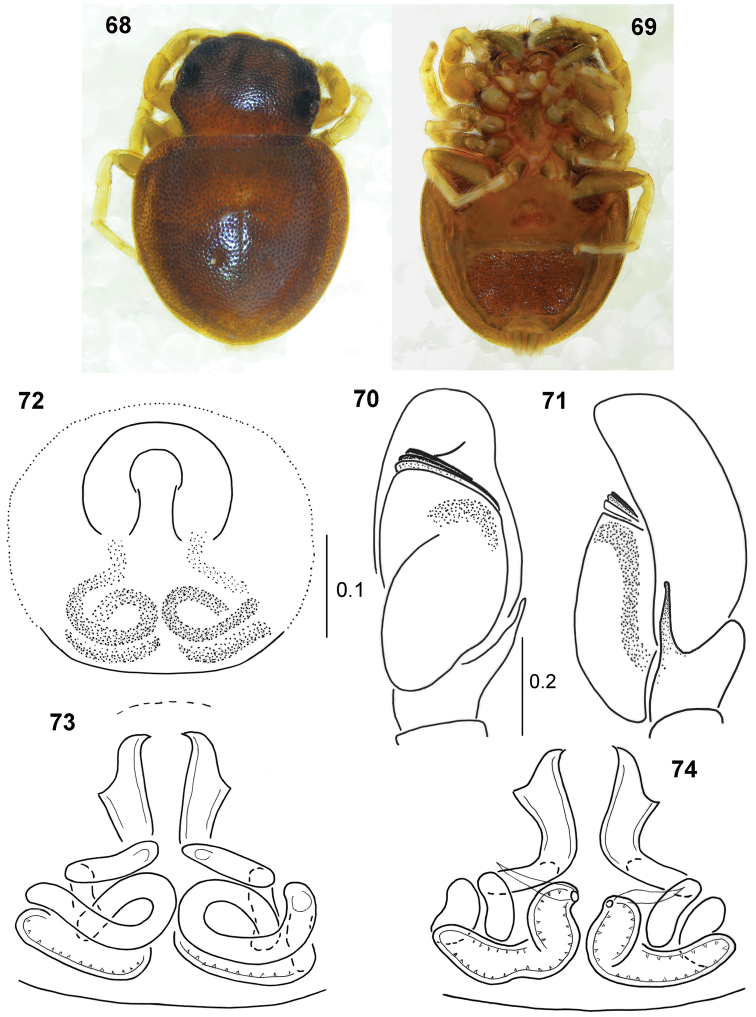
*Pachyballus mombasensis* sp. nov. **68** female, holotype, habitus, dorsal view **69** female, habitus, ventralal view **70** palpal organ, ventral view **71** palpal organ, lateral view **72** holotype, epigyne **73** internal structure of epigyne, ventral view **74** internal structure of epigyne, dorsal view.

#### Etymology.

The specific name is derived from the type locality.

#### Description.

**Male.** Measurements. Cephalothorax: length 1.1, width 1.5, height 0.5. Eye field: length 0.6, anterior width 1.1, posterior width 1.5. Abdomen: length 2.1, width 2.1.

Body flattened, integument strongly sclerotised, clearly pitted. Colouration of carapace dark brown, some bristles by the first row of eyes. Mouth parts brownish with lighter tips. Chelicera with three retromarginal teeth. Abdomen very flat, heart-shaped, dark brown, venter with typical scuta. First pair of legs brown, tibia short, slightly thickened, its dorsal part a little flattened, two pairs of ventral spines and dense dark setae. Other legs yellowish, only femora brownish. Palpal organ as in Figs 70, 71, embolic coil wide and low, tibial apophysis adpressed to cymbium.

**Female.** Measurements. Cephalothorax: length 1.3, width 1.5, height 0.5. Eye field: length 0.7, anterior width 1.1, posterior width 1.5. Abdomen: length 2.1, width 2.1.

General appearance as in Figs 68, 69. Similar to male, first pair of legs not stouter than others, palps dark. Posterior ventral scutum large. Epigyne with semicircular depression (Fig. 72). Internal structure as in Figs 73, 74, copulatory ducts clearly shorter than in congeners, spermathecae comparatively large, strongly sclerotised.

**Immature specimens.** As adults, dorsum of abdomen covered with one large scutum.

#### Distribution.

Known only from the type locality (Fig. 195).

### Pachyballus ornatus sp. nov.

Taxon classificationAnimaliaAraneaeSalticidae

723A6CB3-DF7B-5DB1-8B9C-CAB129ED1025

http://zoobank.org/862AF941-D594-45CC-BBD5-7A38EA629BE7

Figures 2 , 75–80 , 81–85 , 86–91 , 92–97 , 195

#### Holotype.

Tanzania • ♂; Tanga region, Usambara Mts, Amani Nature Reserve, Mbomole Hill; 5°05'S, 38°37'E; 1000 m a.s.l.; 5–8.XI.1995; C. Griswold, N. Scharff, D. Ubick leg.; CAS.

#### Paratypes.

Congo D.R. • 1♂; Equateur, Bokuma; 0°06'S, 18°42'E; in 1952; R.P. Lootens leg.; MRAC 85 126 • 1♂; Mayombe, Bas Congo, Luki Forest Reserve; 5°37'S, 13°05'E; 7.XI.2006; D. De Bakker and J.P. Michiels leg.; primary rainforest, fogging; MRAC 226 115 • 1♂; Kivu, Ibanda [Bukavu]; 2°29'S, 28°50'E; SMF. Tanzania • 2♂ 2♀ (1 of females – black form); together with the holotype; CAS • 1♂; Mufindi distr., Iringa region, Uzungwa Scarp Forest Reserve; 8°32'S, 35°54'E; 750 m a.s.l.; 8.III.1996; McKamey leg.; canopy fogging; UCZM • 1♂; the same locality, by Chita village; 1300–1400 m a.s.l.; 26.X–14.XI.1984; N. Scharff leg.; montane rain forest; UCZM • 3♂ 4♀ (2 of females – black form); 11 km SE Masisiwe Kihanga Stream; 8°32'S, 35°58'E; 1800 m a.s.l.; 17–27V.1997; canopy; UCZM • 1♂; Uluguru Mts, Kimboza Forest Reserve; 7°20'S, 37°47'E; 250 m a.s.l.; 18.VII.1981; M. Stoltze and N. Scharff leg.; UCZM.

**Figures 75–80.**
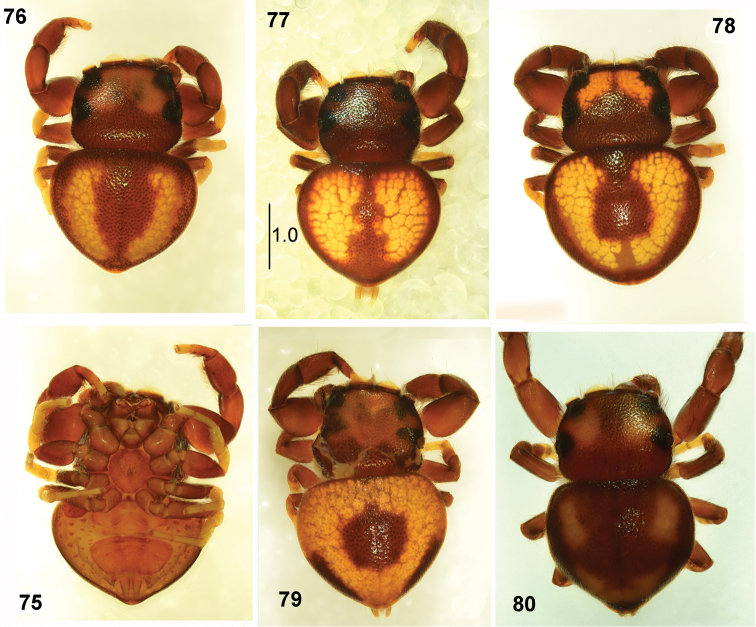
*Pachyballus ornatus* sp. nov., male, habitus **75** ventral view **76–80** dorsal view (all specimens from Tanzania).

#### Diagnosis.

**Figures 81–85.**
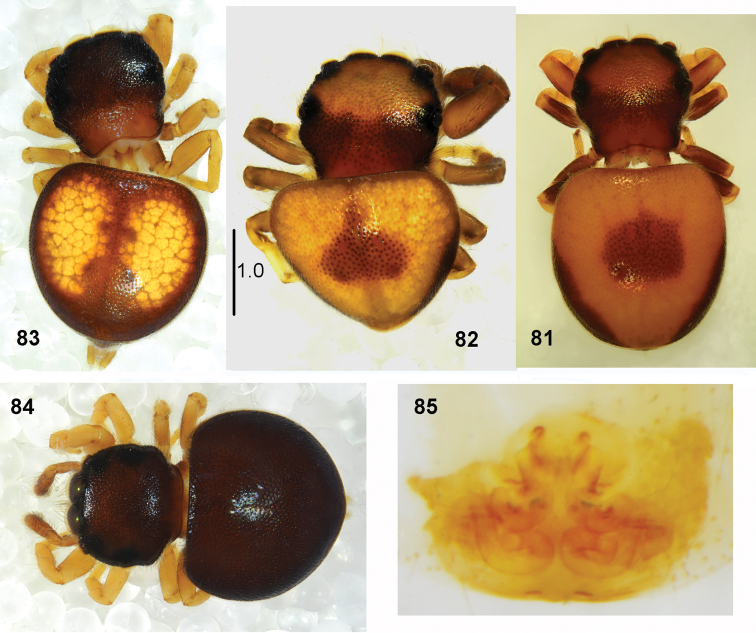
*Pachyballus ornatus* sp. nov., female, **81–84** habitus, dorsal view **85** epigyne (all specimens from Tanzania).

A characteristic body pattern allows an easy recognition of this species; the abdomen is light with dark margins and a dark streak or central patch (Figs 78, 81). Some specimens, probably young adult females (soon after moulting), can be uniformly black and resemble females of *P. flavipes*, but they are distinguishable in having dark palps (that are yellow in the latter species).

#### Etymology.

The specific name is Latin and means *decorated*, which refers to the characteristic colour pattern.

#### Description.

**Male.** Measurements. Cephalothorax: length 1.2–1.7, width 1.5–1.8, height 0.5–0.6. Eye field: length 0.8–1.0, anterior width 1.1–1.4, posterior width 1.5–1.8. Abdomen: length 1.7–2.3, width 1.8–2.4.

**Figures 86–91.**
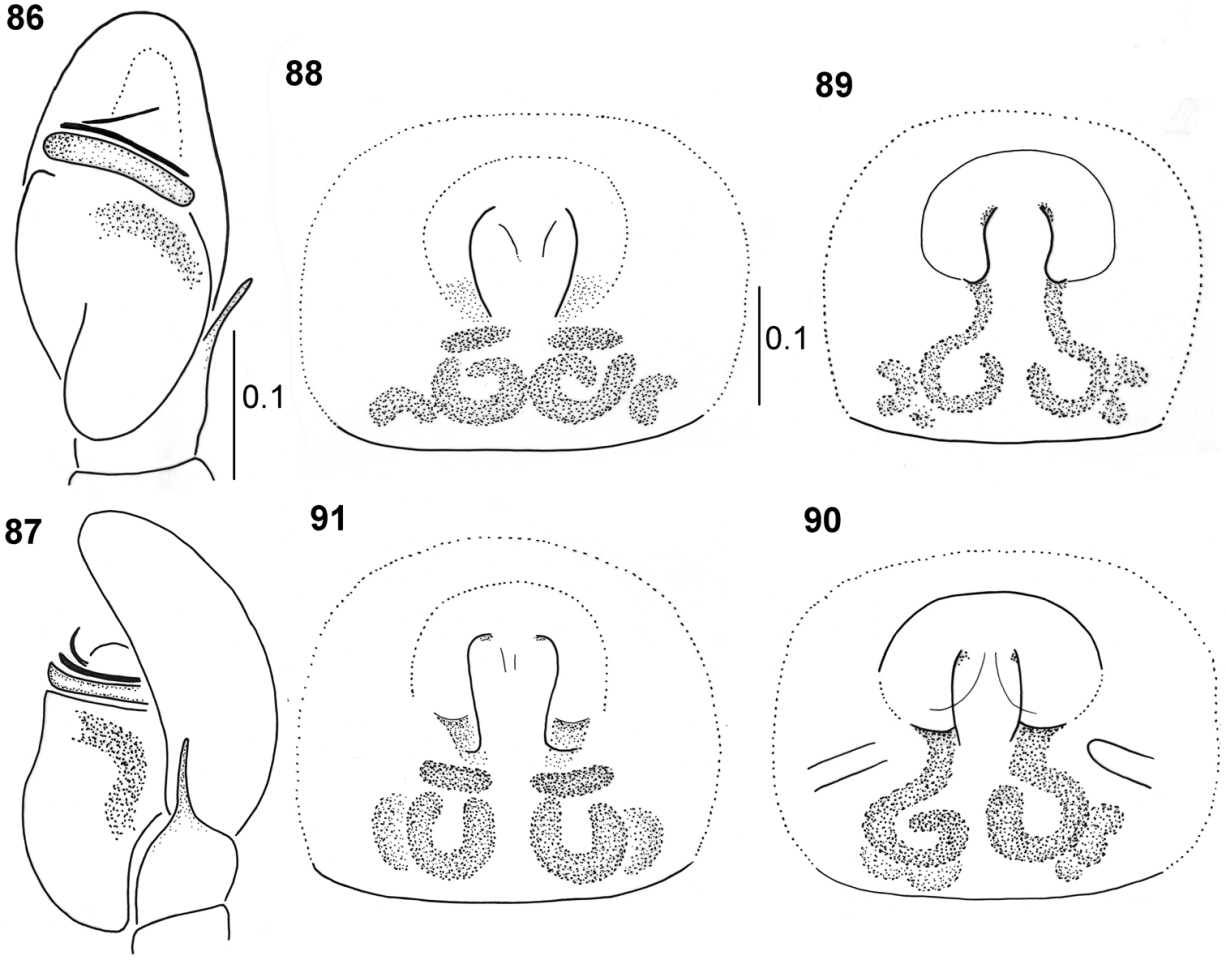
*Pachyballus ornatus* sp. nov., copulatory organs **86** palpal organ, ventral view **87** palpal organ, lateral view **88–91** epigyne (**89, 91** black form **89, 90** specimens from Usambara Mts).

General appearance as in Figs 75–80. Body flat, integument strongly sclerotised, clearly pitted. Carapace wider than long, eye field trapezoid (distance between anterior lateral eyes shorter than between posterior laterals), occupies about half of carapace. Colouration of carapace brown, black around eyes, in some specimens with two large yellowish patches in eye field (Figs 76, 78, 80). Dense bristles at eyes of first row and at lateral edges of carapace. Chelicera with three short teeth on promargin and saw-shaped tooth with four tips on retromargin. Labium and endites brownish with lighter tips, sternum rounded, brown. Abdomen very flat, triangular to heart-shaped, wider than long, its anterior margin almost straight. Colouration of abdomen yellow, brown on the edges, with brown longitudinal median streak (Fig. 77), which is reduced to round patch positioned centrally in some specimens (Fig. 79). Variation of pattern as presented in Figs 76–80. Abdomen with typical ventral scuta (Figs 2, 75). Spinnerets yellow. First pair of legs slightly stouter than other legs, femora enlarged, tibiae slightly thickened, brown, only tarsi light. Other legs yellowish to light brown, femora darker. Palps as in Figs 86, 87, embolic coil low, but basal loop high.

**Female.** Measurements. Cephalothorax: length 1.3–1.5, width 1.4–1.8, height 0.5–0.6. Eye field: length 0.6–0.8, anterior width 1.1–1.2, posterior width 1.4–1.5. Abdomen: length1.9–2.1, width 2.0–2.2.

General appearance as in Figs 81–84. Body shape and colouration similar to that in male. First legs not enlarged. Palps dark. Epigyne with anterior semi-circular depression divided by a median septum (Figs 88–91). Copulatory ducts very long, form several loose loops, spermathecae oval (Figs 92–97).

#### Distribution.

Tanzania and Congo D.R. (Fig. 195).

#### Remarks.

**Figures 92–97.**
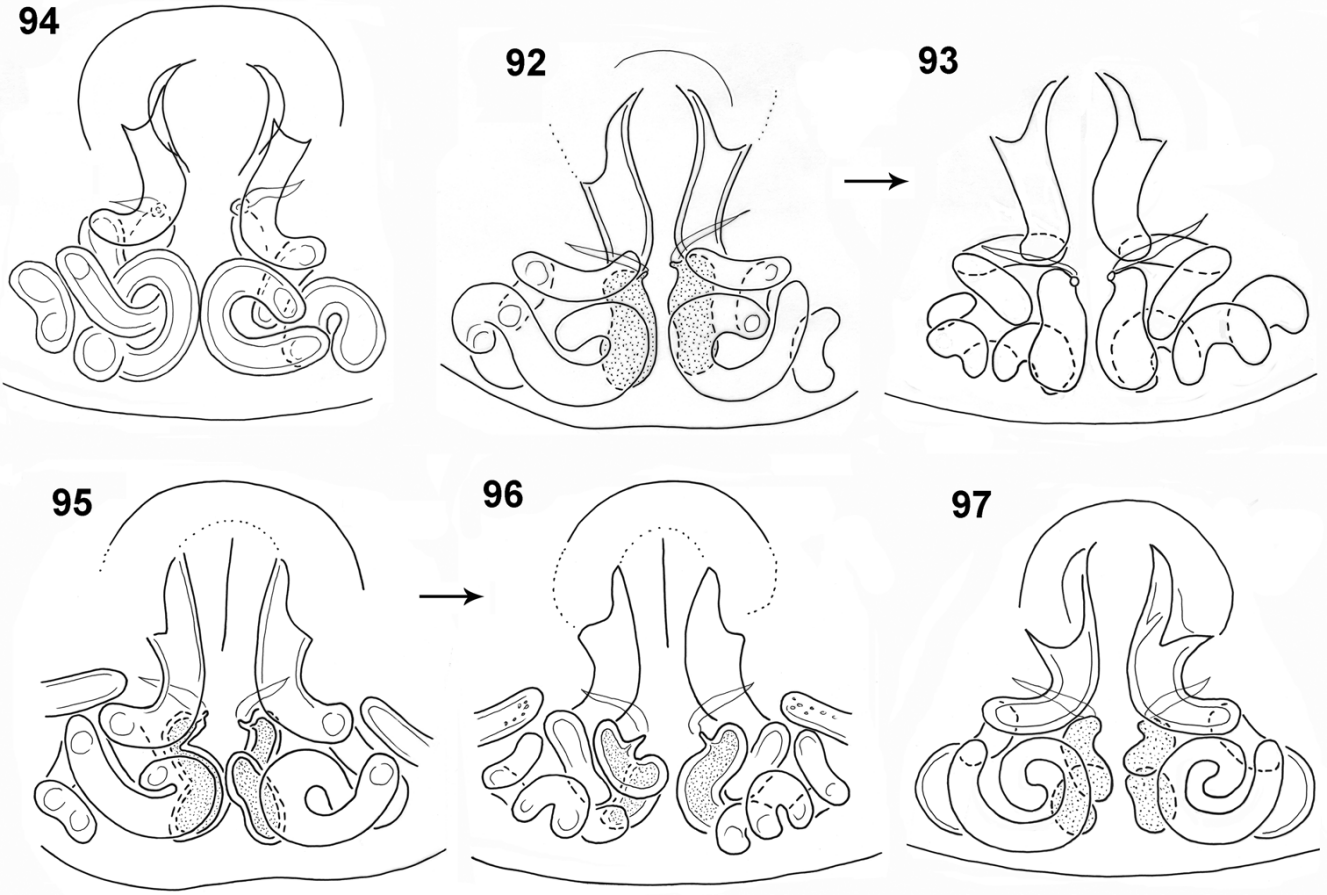
*Pachyballus ornatus* sp. nov., internal structure of epigyne **92, 94, 95, 97** ventral view, **93, 96** dorsal view (specimens **94–96** from Usambara Mts, lower row – black form).

Some specimens, probably young females (soon after the moulting), were dark, almost black (Fig. 84). They appear to become brighter with age due to guanine accumulation (light spots are formed by guanine crystals stored under integument). Black females were collected together with light specimens. Internal structure of epigynes similar in light, Figs 92–94, and black, Figs 85, 95–97 specimens. Colouration seems to turn brighter in the course of life also in males.

### Pachyballus transversus

Taxon classificationAnimaliaAraneaeSalticidae

Simon, 1900

965E3E8E-C29E-53C7-8EC7-E42CDB1FB3CA

Figures 98–103 , 104–109 , 110–116 , 117–122 , 191 , 192 , 196

Pachyballus transversus Simon 1900: 399 (♂); 1901: 482, f. 570–571; 1910: 414; Berland and Millot 1941: 397.

#### Syntypes.

Congo • 2♂; Mayombe; E. Simon coll.; MNHN 7545; examined.

#### Other material examined.

Cameroon • 1♀; Maroua; 10°36'N, 14°19'E; col. C.F. Roewer (nr 12 678); SMF • 1♀; without precise locality; in 1950; J. Birket-Smith leg.; UCZM. Congo D.R. • 1♀; Mayombe, Bas Congo, Luki Forest Reserve; 5°37'S, 13°05'E; 28.IX.2007; D. De Bakker and J.P. Michiels leg.; primary rainforest, fogging; MRAC 226.109A • 3♂ 2 imm; Kivu N prov., Kaisola, Ruindi plain; 0°47'S, 29°17'E; 1100 m a.s.l.; 3.VII.1972; M. Lejune leg.; MRAC 144 487 • 1♂ 2♀ 1 imm.; Ruindi plain, Ndimo Hill; 1100 m a.s.l.; 28.VI.1972; P.M. Lejune leg.; MRAC 144 670 • 1♂; Kivu N prov., Rutshuru; 1°11'S, 29°27'E; III.1937; J. Ghesquière leg.; MRAC 30 583 • 1♂; Goma; 0°34'S, 28°42'E; 10.II.1952; E. Bertrand leg.; MRAC 78 975. Ethiopia • 3♂ 2 imm.; Gorgora; 12°14'N, 37°18'E; in 1961; F. Hartman leg.; MRAC 131 203 • 1♂; Yayu coffe forest plantation; 8°10'N, 36°00'E; in 2004; N. Aklilu leg.; MRAC 231 209 • 1♀; the same locality; beating; MRAC 230 736 • 1 imm.; the same locality; secondary forest, beating; MRAC 229 396. Ivory Coast • 1♀; Bingerville; 5°21'N, 3°54'W; VIII.1962; J. Decelle leg.; MRAC 122 004. Mozambique • 1♀; ‘N Mozambique’; col. C.F.Roewer (nr 9715); SMF. Somalia • 1♀; Sinandogo; in 1946; R. Accigliaro leg.; MRAC 131 175 • 1♀; Giumbo; 0°14'S, 42°37'E; in 1946; R. Accigliaro leg.; MRAC 131 223. Tanzania • 2♂; ‘Zanguebar’ (=eastern coast of tropical Africa, probably Zanzibar); E Simon coll.; MNHN 7021. South Africa • 1♂; KwaZulu-Natal, Ulundi, Ophathe Game Reserve; 28°23'S, 31°24'E; 3.X.2008; C. Haddad leg.; beating, shrubs, overgrazed savanna; NCA 2008/4154 • 1♂; the same locality; 1.X.2008; NCA 2008/3971 • 9♂; the same locality; NCA 2019/1448 • 1♀; Mpumalanga Prov., Nelspruit, Agricultural College; 25°27'S, 30°59'E; 12.XI.1999; P. Stephen leg.; beating, citrus; NCA 2000/223.

**Figures 98–103.**
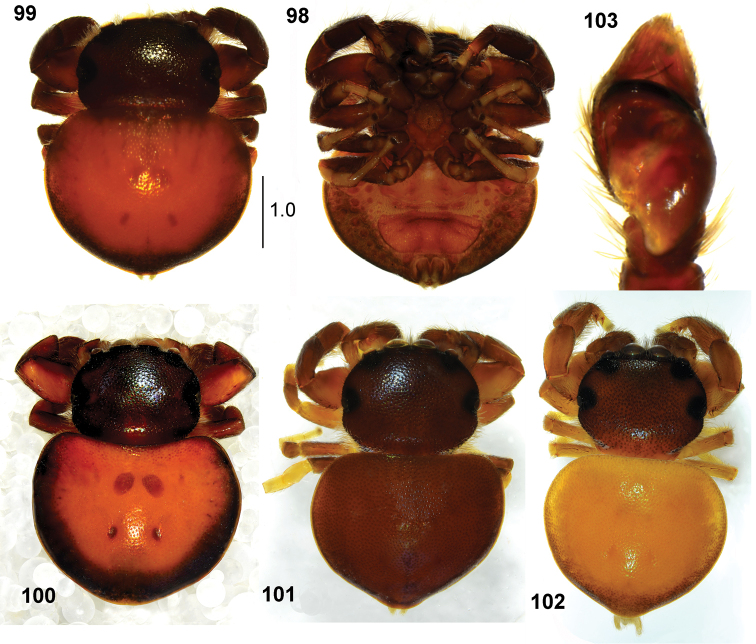
*Pachyballus transversus*, male **98** habitus, ventral view **99–102** habitus, dorsal view, **103** palpal organ, ventral view (all specimens from Congo).

#### Diagnosis.

The body proportions of this species are different than in other *Pachyballus* spp., namely width of carapace and width of abdomen are clearly greater than their length. Sigilla are strongly marked. Shape of the eye field is more trapezoid than in congeners, its width at posterior eye row is a quarter larger than anterior width.

#### Redescription.

**Male.** Measurements. Cephalothorax: length 1.5–1.7, width 1.9–2.0, height 0.6. Eye field: length 0.8–1.0, anterior width 1.3–1.5, posterior width 1.9–2.0. Abdomen: length 2.4–2.9, width 2.8–3.2.

**Figures 104–109.**
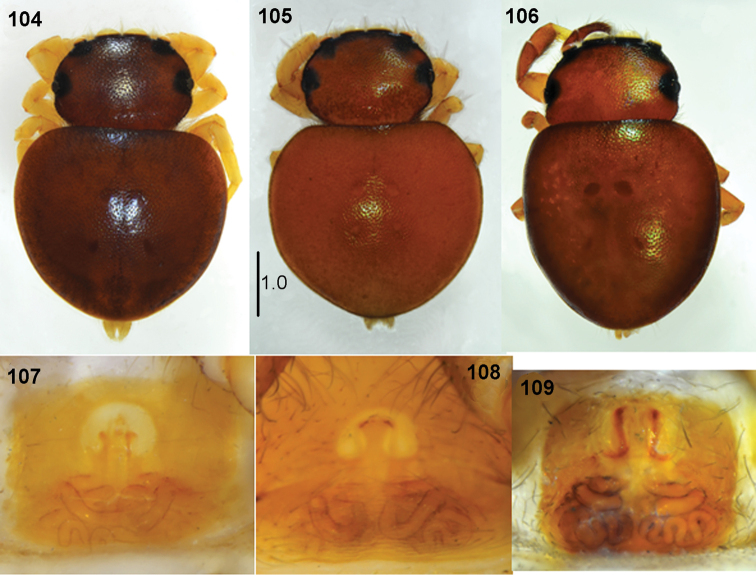
*Pachyballus transversus*, female **104–106** habitus, dorsal view **107–109** epigyne (dissected from specimens visible above) (**104, 106** specimens from Congo **105** specimen from Ethiopia).

**Figures 110–116.**
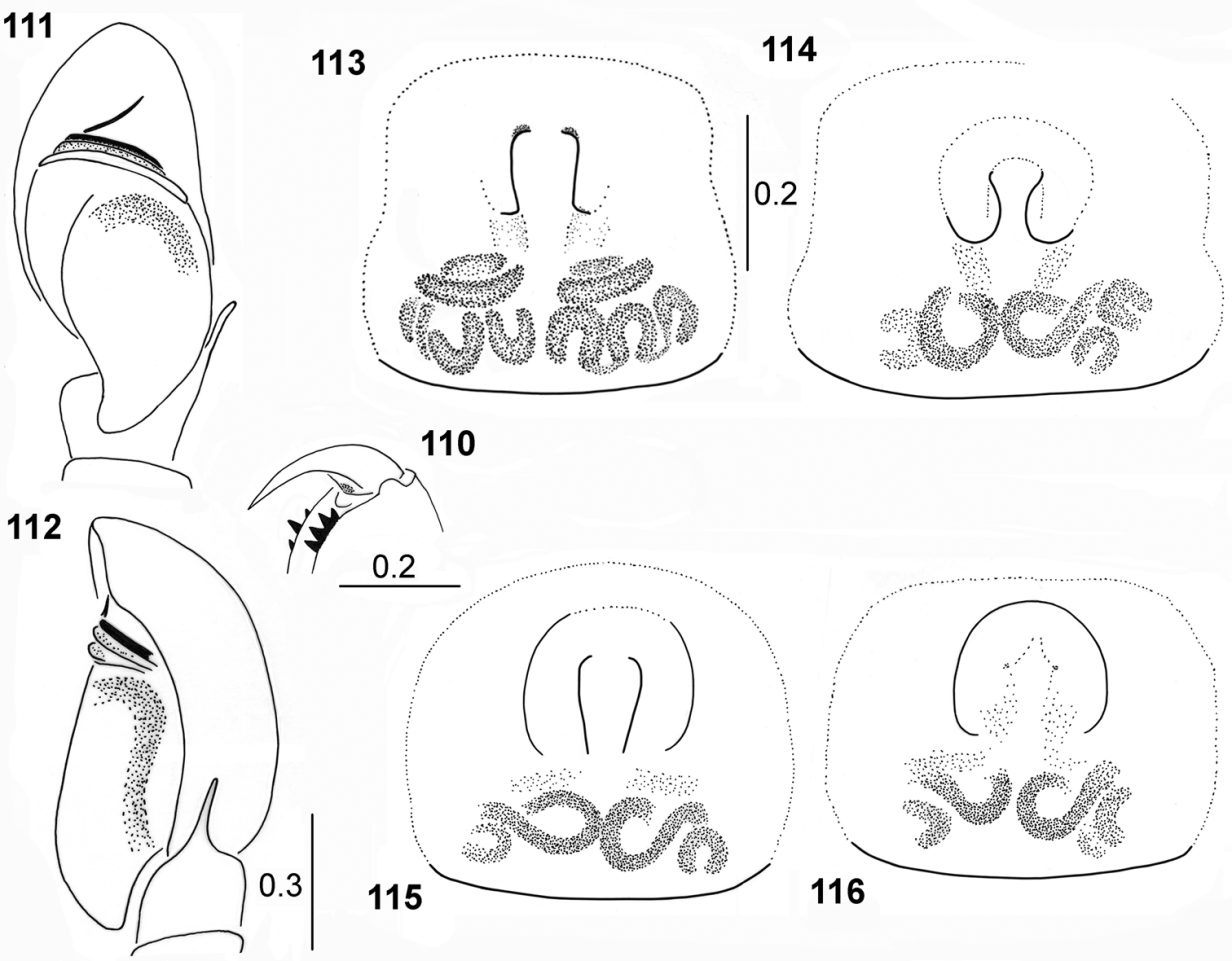
*Pachyballus transversus***110** cheliceral dentition **111** palpal organ, ventral view **112** palpal organ, lateral view **113–116** epigyne (**114** specimen from Ethiopia **115** specimen from Somalia **113, 116** specimens from Congo).

General appearance as in Figs 98–102. Slightly larger than *P. flavipes*, body flattened, covered with hard integument, pitted. Carapace wide, dark brown to blackish, vicinity of eyes black. Eye field strongly trapezoid, more than in other *Pachyballus* spp. Its width at last row of eyes is a quarter larger than its anterior width. A few hairs and long bristles at anterior eyes. Clypeus low. Chelicerae with short fang, three small teeth on promargin and four teeth with fused base on retromargin (Fig. 110). Mouth parts brown, sternum oval, dark. Abdomen heart-shaped, wide, clearly wider than long. Colouration of abdomen usually light, orange brownish with blackish edge, sometimes yellowish or brown. Sigilla clearly visible. Venter brown, with typical scuta (Fig. 98). First pair of legs brown, slightly larger than other pairs, with femora and tibiae thickened, tibiae slightly flattened dorsally. All legs brownish with lighter tarsi. Palps dark, structure as in Figs 103, 111, 112.

**Female.** Measurements. Cephalothorax: length 1.4–1.6, width 1.6–2.0, height 0.7. Eye field: length 0.7–0.8, anterior width 1.2–1.5, posterior width 1.8–2.0. Abdomen: length 2.4–2.9, width 2.8–3.2.

General appearance as in Figs 104–106. Similar to male, eye field strongly trapezoid. Abdomen wide, rounded, with visible sigilla. All legs yellow, sometimes femora, patellae and tibiae brownish. Palps dark. Epigyne typical, with horseshoe-shaped anterior depression (Figs 107–109, 113–116). Copulatory ducts long, forming several loops, spermathecae strongly sclerotised (Figs 118–122).

**Immature specimens.** Shape of body as in adults, abdomen with two oval scuta, close to each other on dorsum (Figs 191, 192). Ventral scuta absent.

#### Distribution.

Widely distributed in Africa (Fig. 196).

#### Remarks.

Simon (1900) described only the female of *P. transversus*. He recorded this species from Congo, Transvaal (South Africa) and Zanzibar (Simon 1900, 1901). He found it also (Simon 1910) later in Guinea Bissau and noted in this publication that this species had been described from Congo. This suggests that the Congolese specimens constitute the “type material”. However, the two specimens from Congo, personally labelled by Simon and kept in the MNHN collection, are males. The fact was also mentioned by Berland and Millot (1941). Simon most probably described these specimens and apparently published the wrong data on sex of specimens. This description is very superficial and the structure of copulatory organs is not depicted. There are also some other males from Zanzibar in Simon’s collection in MNHN (but no female). Simon’s specimens from Natal, kept in MZC (examined) are immature and probably misidentified. Thus, it should be concluded that Simon’s description concerned the male and the female of this species is described here for the first time. The figure in Simon (1901) shows characteristic body proportions of *P. transversus* (width of carapace greatly exceeds its length, eye field is clearly trapezoid with long distance between eyes in the posterior row, and abdomen is rounded), which allows the proper species recognition.

**Figures 117–122.**
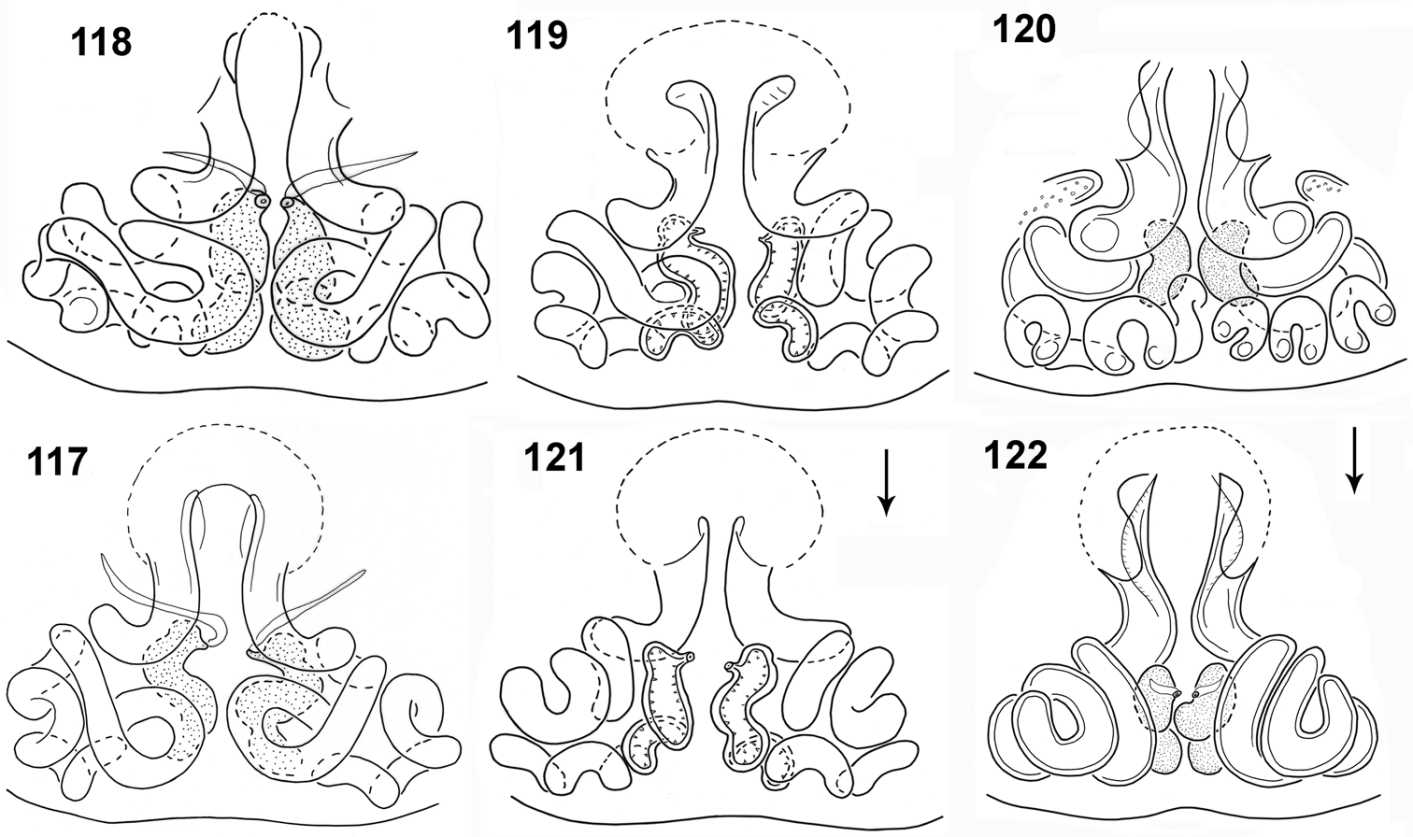
*Pachyballus transversus*, internal structure of epigyne **117–120** ventral view **121, 122** dorsal view (**117** specimen from Ethiopia **118** specimen from Somalia, other specimens from Congo).

### Pachyballus variegatus

Taxon classificationAnimaliaAraneaeSalticidae

Lessert, 1925

38FCB30A-9569-5A5A-838A-F60C19D7C421

**Figures 123–128.**
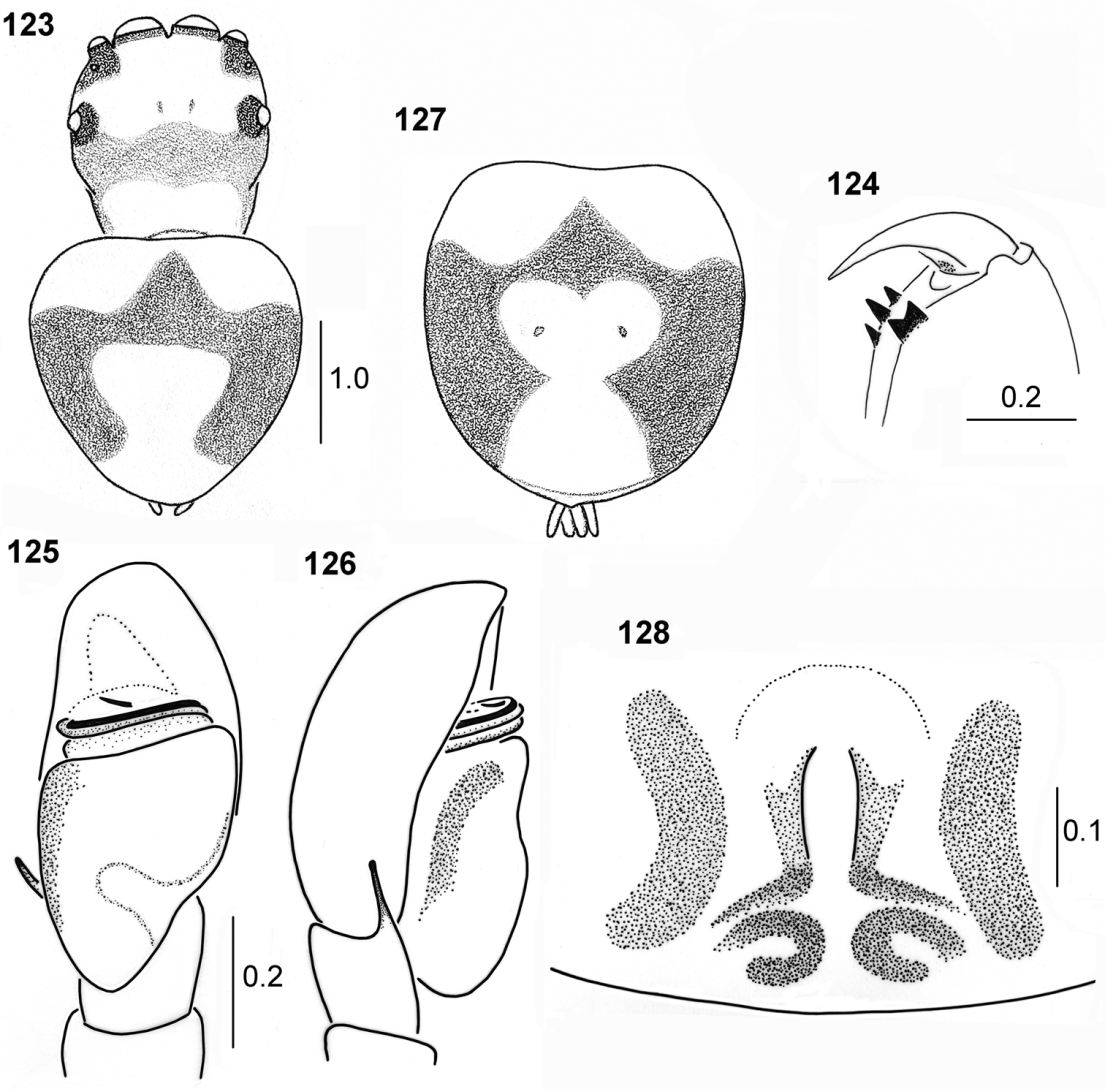
*Pachyballus variegatus*, syntypes **123** male, habitus**124** cheliceral dentition **125** palpal organ, ventral view **126** palpal organ, lateral view **127** female, colouration of abdomen **128** epigyne.

Figures 123–128 , 193

Pachyballus variegatus Lessert 1925: 437, f. 10–14 (♂♀).

#### Syntypes.

Tanzania • 1♂ 1♀; Kilimanjaro, Kibonoto; 3°11'S, 37°06'E; Sjöstedt leg.; NHRS; examined.

#### Diagnosis.

This species is the only one in the genus that does not have the posterior ventral scutum on abdomen. Its other diagnostic feature is the pattern on abdomen; the anterior one-fourth of the abdomen is bright, the posterior part dark with a wide, light median patch (Figs 123, 127).

#### Redescription.

**Male.** Measurements: Cephalothorax: length 1.4, width 1.3, height 0.4. Eye field: length 0.7, anterior width 1.1, posterior width 1.3. Abdomen: length 1.8, width 1.7.

General appearance as in Fig. 123. Body flattened, integument strongly sclerotised, clearly pitted. Eye field light, yellowish orange, blackish rings around eyes, thoracic part light brown anteriorly, posterior slope yellowish. A few delicate hairs at anterior eyes. Chelicerae brown, with three teeth on promargin and a tooth with two tips on retromargin (Fig. 124). Labium and endites basally brownish, with light tips. Sternum light brown. Abdomen heart-shaped, widest anteriorly and tapering, anteriorly orange, sides brown with wide orange area in the middle (Fig. 123). Venter yellowish, without scutum. Spinnerets short, yellow. Legs relatively short, dark yellow (first pair absent in the syntype, only coxae extant, brown). Pedipalp (only right present) brown. Palpal organ as in Figs 125, 126, embolic coil wide, with three loops.

**Female.** Measurements: Cephalothorax: length 1.5, width 1.4, height 0.5. Eye field: length 0.8, anterior width 1.2, posterior width 1.4. Abdomen: length 2.2, width 2.1.

Colouration as in male (Fig. 127). Abdomen rounded. Palp light, with brown apical part. Epigyne weakly sclerotised with shallow central depression (Fig. 128). Internal structure not studied, spermathecae probably large (visible through integument).

#### Remarks.

Lessert (1925) wrote in the original description that the basic colour of body was black. The specimens must have bleached heavily, however the outline of lighter patches has been preserved.

#### Distribution.

Known only from the type locality (Fig. 193).

### Peplometus

Taxon classificationAnimaliaAraneaeSalticidae

Simon, 1900

23F14A91-6ED9-5D79-B7EE-A6992F837F9D

Peplometus Simon 1900: 399; 1901: 486.

#### Type species.

*Homalattus biscutellatus* Simon, 1887.

#### Diagnosis.

*Peplometus* is closely related to *Pachyballus*. From the latter genus it can easily be separated by the elongated abdomen (rounded in *Pachyballus*). It differs also in the form of leg I in males having the leaf-like setae on tibia I (absent in the other genus).

#### Description.

Small spiders (ca. 3.0–4.0 mm length), with very flat body, covered with hard strongly sclerotised integument. Carapace almost square-shaped, pitted dorsally, its length only slightly exceeds its width. Chelicerae with two (exceptionally three) small teeth on promargin and on retromargin a saw-shaped tooth with four to five denticles. Abdomen slightly elongated, ratio of its length to width 1.3–1.5 (only in female of *P. biscutellatus* 1.2). In males, width of abdomen almost equal to width of carapace, abdomen of females wider and rather oval. Anterior margin of abdomen straight. Hard scutum covers dorsum of abdomen, venter also strongly armoured, with scuta as in *Pachyballus*, namely the narrow scutum with backwards extending “horns” at anterior edge, and a large trapezoid scutum in posterior half of abdomen. Males with a thinner sclerotised plate in front of epigastric furrow, its thick posterior margin forms narrow wedge-shaped bar (Figs 138, 185). Numerous sclerotised bumps on sides of the abdominal venter (Fig. 138). Legs short, in males the first pair slightly thicker, with enlarged femora and modified tibiae. In some species conspicuously thickened, usually flattened, in other species elongated (in this case the metatarsus I with a dorsal process). Tibiae I black, with ventral brush of long dense flattened setae, contrasting with other segments. Copulatory organs similar to those in *Pachyballus*.

**Figures 129–135.**
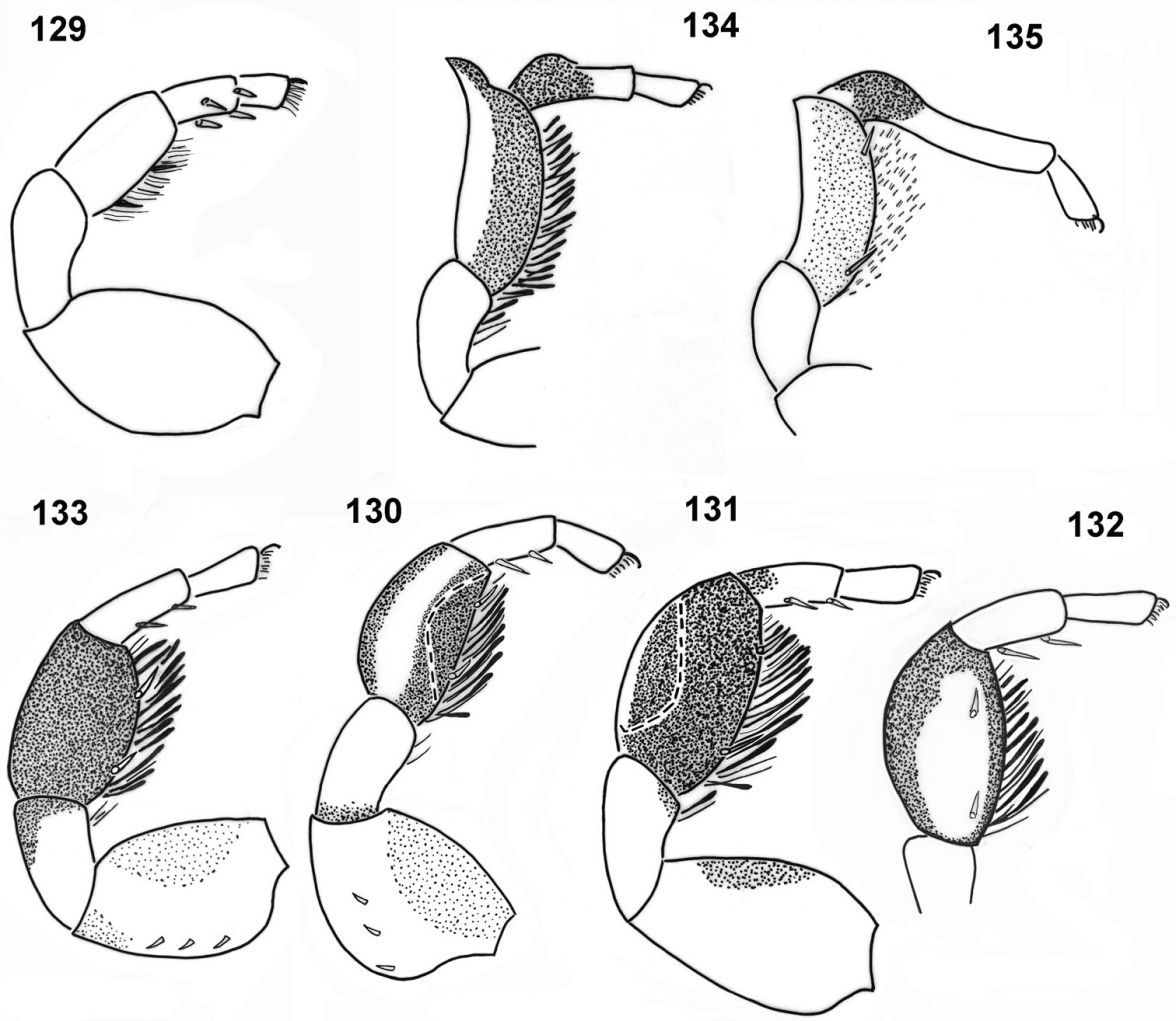
First leg of male **129***Pachyballus castaneus***130***Peplometus biscutellatus***131, 132***P. chlorophthalmus***133***P. congoensis***134***P. oyo***135***P. nimba***132** retrolateral view, others prolateral view.

### Key to *Peplometus* males

**Table:** 

1	Metatarsus I with a dorsal process, tibia not thickened	**2**
–	Metatarsus I without a process, tibia strongly thickened	**3**
2	Tibia I with a distal process, metatarsus shorter than tibia (Fig. 134)	***P. oyo***
–	Tibia I without process, length of metatarsus equal to length of tibia (Fig. 135)	***P. nimba***
3	Tibia I not flattened (Fig. 133)	***P. congoensis***
–	Tibia I flattened	**4**
4	Tibia I flattened dorsally (Fig. 131)	***P. chlorophthalmus***
–	Tibia I flattened prolaterally (Fig. 130)	***P. biscutellatus***

### Peplometus biscutellatus

Taxon classificationAnimaliaAraneaeSalticidae

(Simon,1887)

32E07CB6-B301-510D-BD8D-9CB4600EF047

Figures 4 , 130 , 136–142 , 143–145 , 146–149 , 150–153 , 189 , 190 , 197

Homalattus biscutellatus Simon 1887: 263 (♀).
Peplometus biscutellatus Simon 1901: 486; Berland and Millot 1941: 398.

#### Holotype.

Ivory Coast • ♀; Assinie; 5°08'N, 3°16'W; Alluaud [C.] leg.; MNHN 9072; examined.

#### Other material examined.

Cameroon • 2♀; Matube, Tiko plantation; 4°04'N, 9°21'E; 24.IV-6.V.1949; B. Malkin leg.; CAS • 1♀; Mabete Victoria [Limbe]; 4°01'N, 9°13'E; 24.V-7.VI.1949; B. Malkin leg.; CAS • 1♀; Reserve Forestiere Nyong, S to Makak; 3°25'N, 12°47'E; 17.II.1950; J. Dahl and J. Birket-Smith leg.; UCZM. Ghana • 2♂; Kakum forest; 5°25'N, 1°19'W; 18.XI.2005; primary forest, R. Jocqué, D. De Bakker and L. Baert leg.; fogging; MRAC 217 897 • 1♀; the same data; 16.XI.2005; MRAC 217 875 • 2♀; the same data; 25.XI.2005; MRAC 217 945 • 7♂ 4♀; the same locality; 15.XI.2005; secondary forest; MRAC 217 865 • 2♂; the same data; 17.XI.2005; MRAC 217 881 • 1♂; the same data; 12.XI.2005; MRAC 217 857. Guinea • 1♀; Dalaba; 10°42'N, 12°15'W; VII.1937; J. Millot leg.; MNHN • 1♂; Nimba Mts, Zougué Valley, near Gbakoré mine camp; 7°34'N, 8°28'W; 5.X.2011; 780 m a.s.l.; young secondary gallery forest, fogging canopy; D. van den Spiegel leg.; MRAC 238 187 • 1♂; same locality, Seringbara road; 8.II.2012; beating; A. Henrard, C. Allard, P. Bimou, M. Sidibé leg.; MRAC 239 050 • 2♂, 1♀; same locality; gallery forest of Zié; 3.X.2011; 1250 m a.s.l.; fogging, canopy forest, understory shrub layer; D. van den Spiegel, A. Hernard leg.; MRAC 238 066. Ivory Coast • 1 imm.; together with the holotype • 1♂; Bouaké, Foro Foro; 7°49'N, 5°01'W; 5–7.VIII.1974; pitfall; G. Couturier leg.; MRAC 216 442 • 1♂; the same data; MRAC 216 380. Nigeria • 1♂; Kabba; 7°50'N, 6°04'E; 18–23.II.1949; B. Malkin leg.; CAS. Senegal • 1♂; Dakar; 8°19'N, 0°13'W; IX.1947; L. Berland leg.; MNHN. Sierra Leone • 2♂ 3♀ 2 imm.; Free Town; 8°30'N, 13°15'W; col. E. Simon, MNHN 19 988. Togo • 1♀ 1 imm.; without precise locality; col. C.F. Roewer (nr 10861); SMF.

**Figures 136–142.**
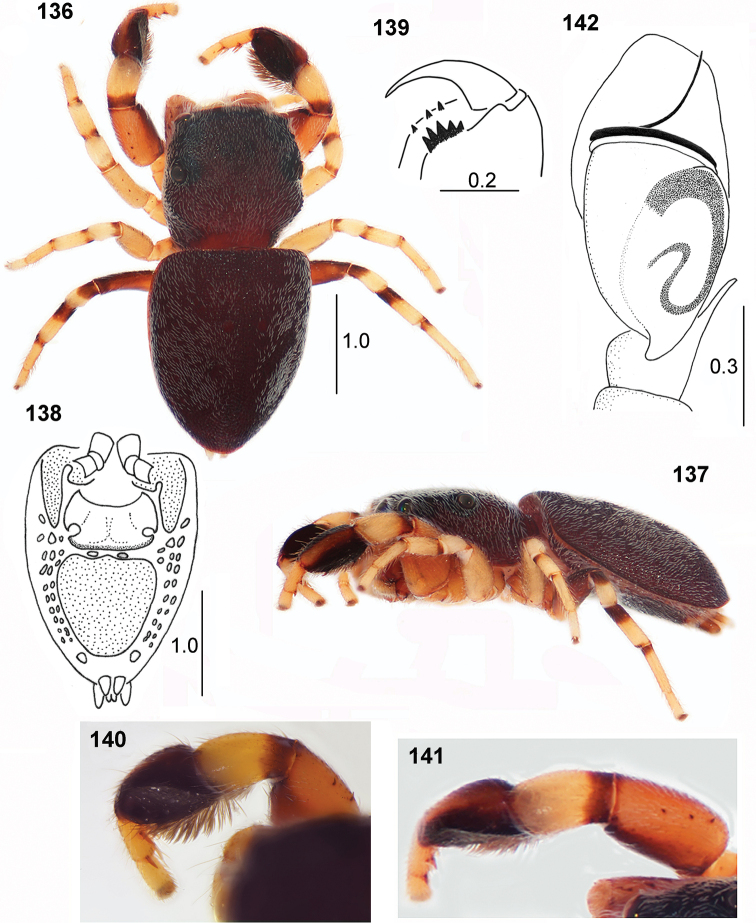
*Peplometus biscutellatus*, male **136** habitus, dorsal view **137** habitus, lateral view **138** venter of abdomen **139** cheliceral dentition **140** first leg, dorsoprolateral view **141** first leg, dorsal view **142** palpal organ, ventral view (photos – specimen from Ghana, drawings – specimen from Sierra Leone).

#### Diagnosis.

The male of this species may be distinguished by a flat area on prolateral side of the tibia I (Figs 130, 140, 141). The female has a cordiform abdomen, while other congeners have oval abdomen; the width/length ratio of abdomen in *P. biscutellatus* female is 0.8, whereas it is 0.75 in other species.

#### Redescription.

**Male.** Measurements. Cephalothorax: length 1.1–1.7, width 1.2–1.8, height 0.5–0.6. Eye field: length 0.7–0.9, anterior width 1.0–1.3, posterior width 1.2–1.6. Abdomen: length 1.9–2.9, width 1.3–2.1.

**Figures 143–145.**
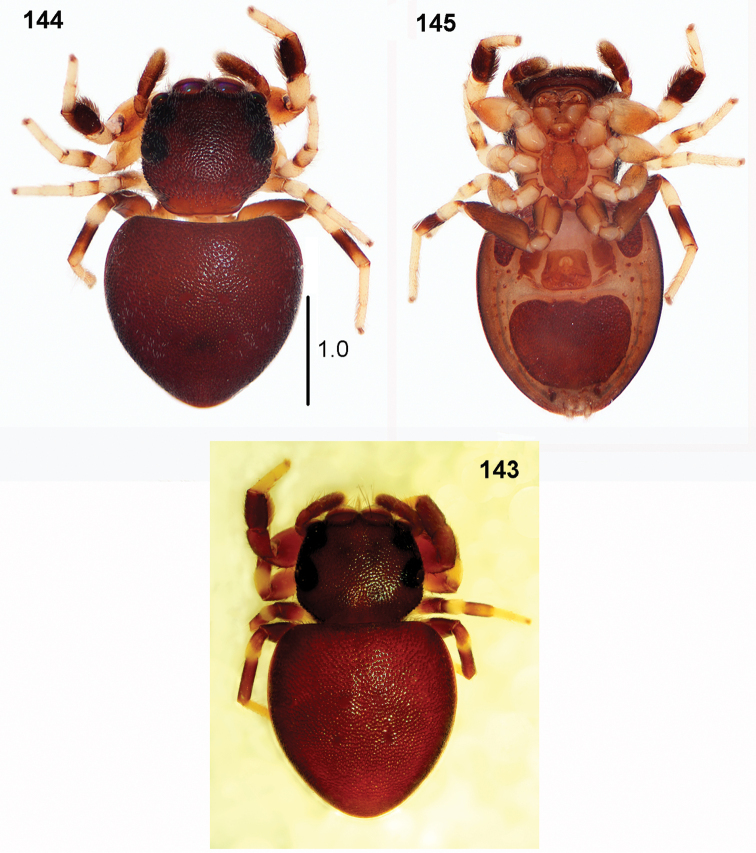
*Peplometus biscutellatus*, female **143, 144** habitus, dorsal view **145** habitus, ventral view (**143** specimen from Cameroon **144, 145** specimen from Ghana).

General appearance as in Figs 136, 137. Body flattened, completely covered with strongly sclerotised and pitted integument. Dorsum of body dark brown, clothed in dense short white hairs (lost in some of the studied specimens). Eye field on more than half of carapace length, black rings around eyes, the first row of eyes encircled with white hairs. Long white bristles between anterior eyes. Clypeus extremely low, brown with a few white hairs. Chelicerae with three diminutive teeth on promargin and five retromarginal teeth fused basally (Fig. 139). Mouthparts brown. Sternum oval, brown. Abdomen elongated, widest anteriorly and tapering rearward, anterior edge almost straight. Dorsum of abdomen totally covered by very strongly sclerotised and pitted scutum, laterally slightly turned back to venter. Venter with typical scuta; anterior scutum narrow, extending backwards laterally, posterior ventral scutum largest, cordiform. In front of epigastric furrow a thin sclerotised plate with thick posterior margin forms narrow wedge-shaped bar. Numerous bumps on sides ventrally; armour-plate pattern is shown in Fig. 138. Spinnerets brown, obscured by dorsal scutum. Legs I the biggest, femora enlarged, brownish with darker prolateral side, three short spines on dorsum. Tibia thickened, clearly flattened prolaterally, blackish with pale dorsal streak, ventrally black brash of dense long feather-shaped setae (Figs 130, 140), metatarsi and tarsi short, yellowish, two pairs of ventral spines on metatarsi. Other legs pale yellowish with dark marks at bases of segments, only femora IV brown (or yellow with brown streak on prolateral side). Palps light, their structure as in Fig. 142. Palpal tibia short with single thin straight apophysis, bulb oval, embolus thin, long, spirally coiled, forming three loops on bulb tip.

**Female.** Measurements: Cephalothorax: length 1.0–1.4, width 1.2–1.4, height 0.5–0.6. Eye field: length 0.6–0.8, anterior width 1.0–1.2, posterior width 1.2–1.3. Abdomen: length 2.1–2.3, width 1.7–1.9.

**Figures 146–149.**
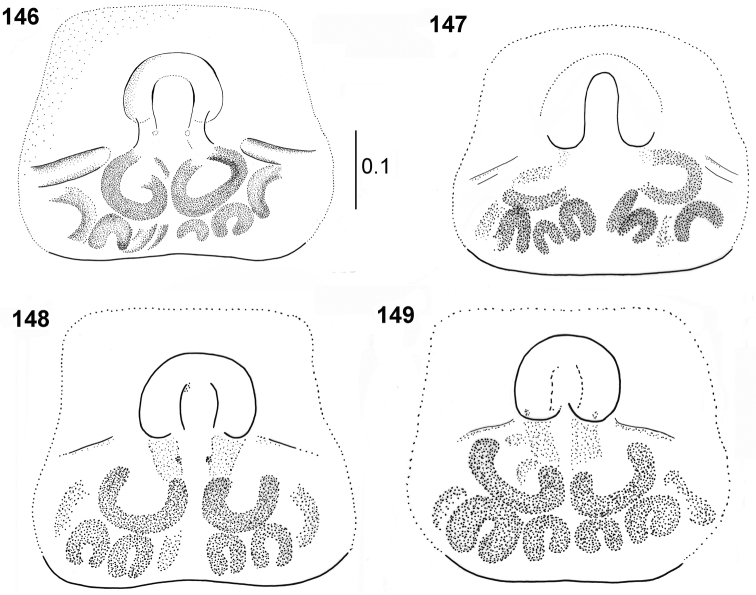
*Peplometus biscutellatus*, female, epigyne (upper row specimens from Sierra Leone, lower row specimens from Ghana).

**Figures 150–153.**
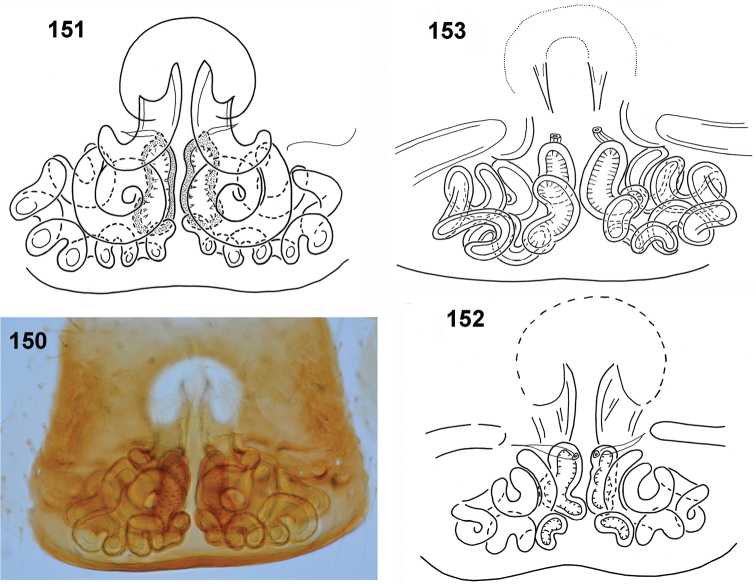
*Peplometus biscutellatus*, female, internal structure of epigyne **150, 151** ventral view **152, 153** dorsal view (**150, 151** specimen from Ghana **152** specimen from Cameroon **153** specimen from Sierra Leone).

General appearance as in Figs 143–145. Similar to male, but abdomen wider, heart-shaped. Scarce white hairs on body. Carapace dark brown, black near eyes. Legs I as in males, not so thick, tibia slightly thickened, retrolateral flattening hardly visible. Palps dark, blackish. Epigyne large, rectangular, weakly sclerotised, with small semicircle shallow depression divided by median septum (Figs 146–149). In some females horizontal crevices laterally, formed by microsculpture of integument, not part of epigyne (Fig. 146). Copulatory ducts very long, spirally coiled behind openings, form several loops distally, spermathecae bean-shaped (Figs 150–153).

**Immature specimens.** Abdomen not elongated, heart-shaped, with two oval dorsal scuta on abdomen, close to each other (Figs 189, 190).

#### Remarks.

The first description of the male is given here. Simon described *P. biscutellatus* based on a female from Ivory Coast, however in his samples from Sierra Leone the two sexes were present (labelled by Simon himself). This fact has already been mentioned by Berland and Millot (1941), but they have not described the missing male. Material collected by Simon is in a very poor condition. Numerous specimens have lately been collected by fogging, this species typically inhabits canopy.

#### Distribution.

West equatorial Africa (Fig. 197).

### Peplometus chlorophthalmus

Taxon classificationAnimaliaAraneaeSalticidae

Simon, 1900

C5D685B8-71AB-5540-BB1E-9FFFFCFF1B38

Figures 131 , 132 , 154–159 , 160–165 , 166–171 , 197

Peplometus chlorophthalmus Simon 1900: 399 (♂); 1901: 482, f. 566–569.

#### Holotype.

South Africa • ♂; Natal (eastern SA); C. M[artin] [leg.]; MNHN 17 385; examined.

#### Other material examined.

South Africa • 1♀ 5 imm.; together with the holotype. Congo D.R. • 1♂ 1♀ 1 subad. ♂; Bas Congo, Mayombe, Luki Forest Biosphere Reserve; 5°40'S, 13°10'E; 7.XI.2006; D. De Bakker and J.P. Michiels leg.; beating; MRAC 221 505 • 1♀; the same data; 14.XI.2006; MRAC 219 997 • 1♀; the same data; 8.XI.2006; MRAC 219 944 • 2♀; the same data; 14.XI.2006 and 19.IX.2007; MRAC 226 100 • 1♂ 1♀; the same locality; 24.IX.2007; old secondary rainforest, fogging; MRAC 226 104 • 1♀; the same locality; 29.IX.2007; primary rainforest, fogging; MRAC 226 110 • 2♀; the same data; 5.XI.2006; MRAC 226 118 •1♀; the same data; 11.XI.2006; MRAC 221 583 • 1♀; the same data;12.XI.2006; MRAC 220 954.

**Figures 154–159.**
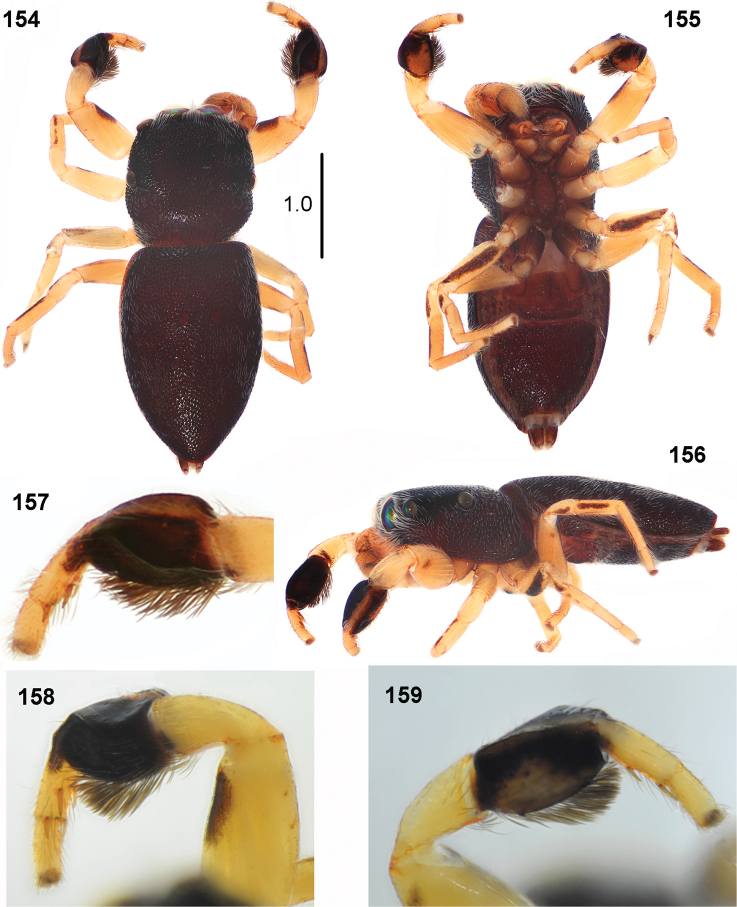
*Peplometus chlorophthalmus*, male **154** habitus, dorsal view **155** habitus, ventral view **156** habitus, lateral view **157** first leg, dorsal view **158** first leg, prolateral view **159** first leg, retrolateral view (specimen from Congo).

#### Diagnosis.

The tibia of the leg I in male is strongly flattened dorsally (Figs 131, 158). The female is difficult to distinguish from *P. biscutellatus*, but it has a narrower abdomen (see Diagnosis of the latter species).

#### Redescription.

**Male.** Measurements. Cephalothorax: length 1.0–1.5, width 1.1–1.3, height 0.5–0.6. Eye field: length 0.6–0.8, anterior width 1.0–1.2, posterior width 1.2–1.3. Abdomen: length 2.2–2.3, width 1.4–1.5.

**Figures 160–165.**
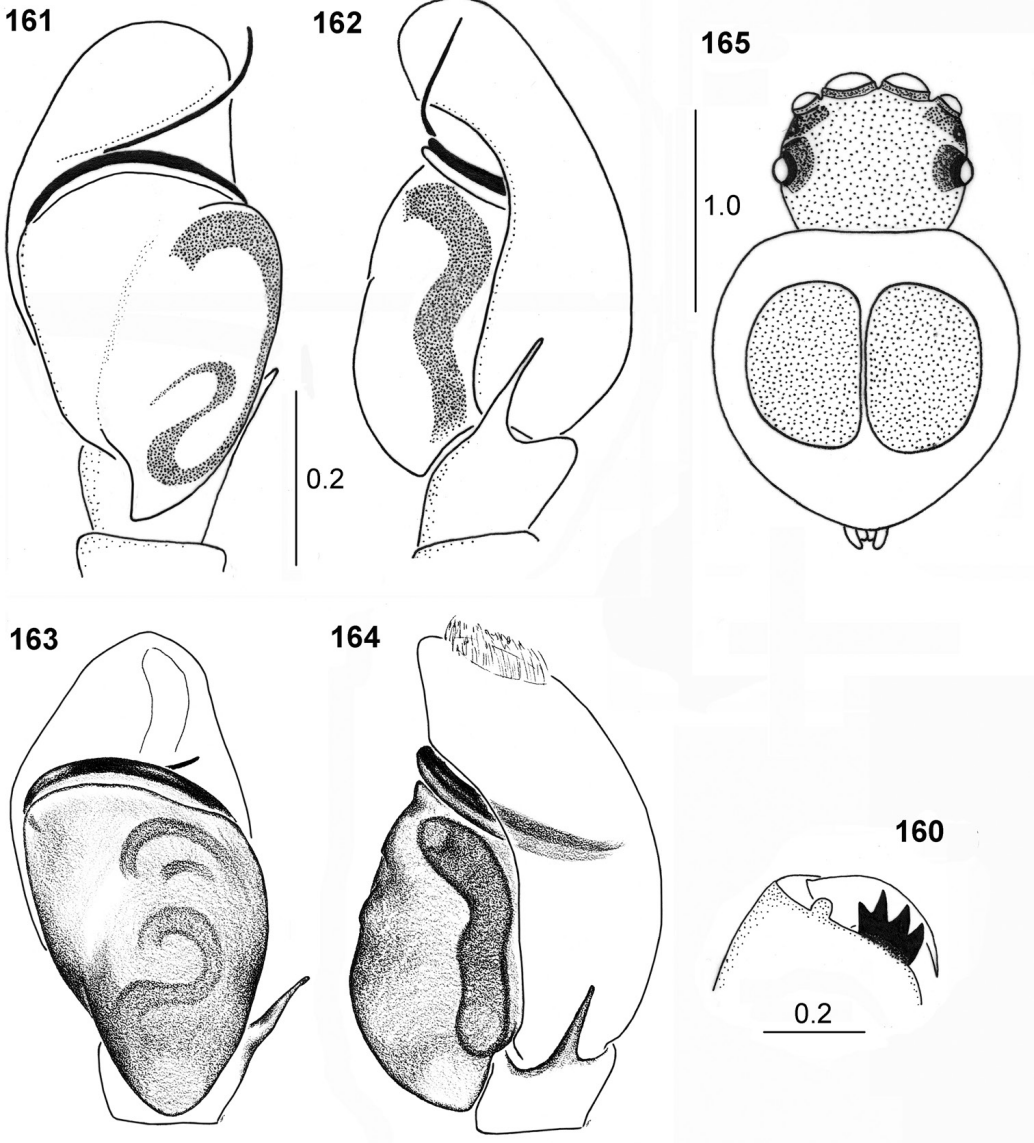
*Peplometus chlorophthalmus***160** cheliceral dentition **161, 163** palpal organ, ventral view **162, 164** palpal organ, lateral view **165** immature specimen (**160–162** holotype **163, 164** specimen from Congo).

General appearance as in Figs 154–156. Small spider with flattened body. Carapace dark brown to blackish, pitted. White bristles around anterior median eyes and between all eyes of first row. Clypeus low, black, with a few white hairs. Fang of chelicerae short, two small teeth on promargin, retromarginal tooth with four tips (Fig. 160). Sternum oval, dark brown. Abdomen narrow, covered with strongly sclerotised, pitted, dark integument. Venter with typical scuta (same as by *P. biscutellatus*). Legs yellowish with dark lines along femora and tibiae III and IV on prolateral surface. Legs I the stoutest, tip of prolateral side of femur with small patch, tibia strongly thickened, black with yellow line along dorsum and large light patch on retrolateral side, long flattened black setae ventrally (Figs 157, 159). Tibia I characteristic for having a large flattened part on dorsal side (Figs 131, 158). Pedipalp light, its structure as in Figs 161–164, embolic coil wide.

**Female.** Measurements. Cephalothorax: length 1.0–1.4, width 1.1–1.3, height 0.5–0.6. Eye field: length 0.6–0.7, anterior width 1.0–1.2, posterior width 1.2–1.3. Abdomen: length 2.0–2.4, width 1.5–1.8.

**Figures 166–171.**
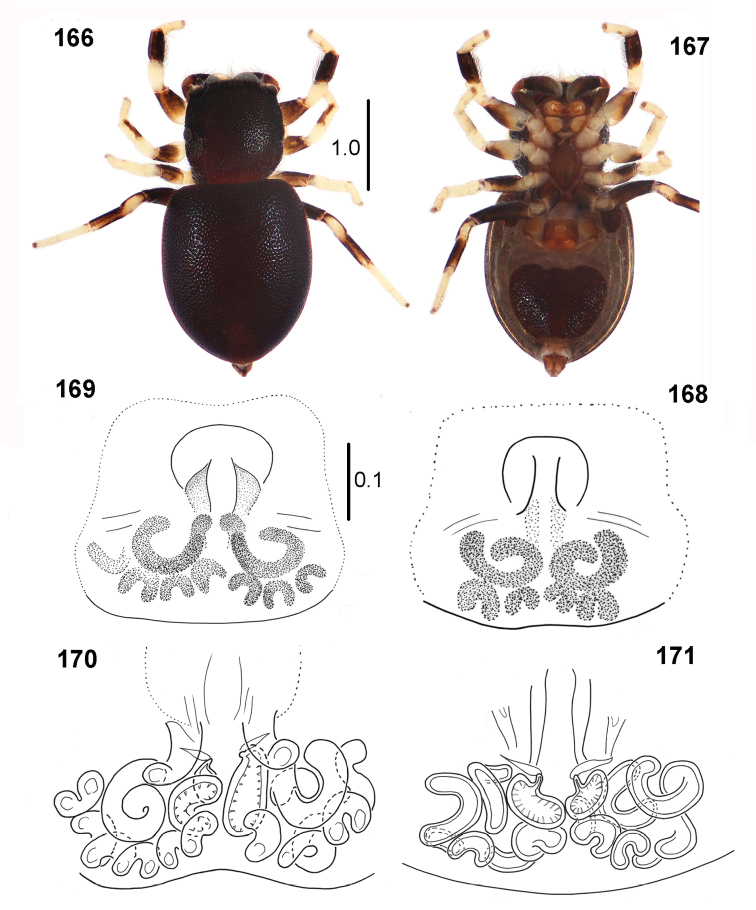
*Peplometus chlorophthalmus*, female **166** habitus, dorsal view **167** habitus, ventral view **168, 169** epigyne **170** internal structure of epigyne, ventral view **171** internal structure of epigyne, dorsal view (**171** specimen from Natal, other specimens from Congo).

Similar to male, general appearance as in Figs 166, 167. White hairs at anterior eyes and on posterior carapace slope. Abdomen wider than in male, but relatively narrow, narrower than in *P. biscutellatus*. Legs darker than in male. Femora of I–III legs brown with yellow ventral surface, femora IV completely brown. Patellae IV with pro- and retrolateral brown stripes. First leg not larger, its tibia black with black long setae ventrally, in some specimens with narrow light streak along dorsum. Tibiae II with prolateral brown stripe, tibiae IV brown with thin yellow longitudinal stripes dorsally and ventrally. Other leg segments light yellow. Palps blackish. Epigyne as in Figs 168, 169, rectangular with shallow depression. Ventral structure of epigyne similar to other species, copulatory ducts long, weakly sclerotised in initial part, forming several loops (Figs 170, 171).

**Immature specimens.** Abdomen not elongated, heart-shaped, with two oval scuta on dorsum, close to each other, not covering whole dorsum of abdomen (Fig. 165).

#### Remarks.

The first description of the female is given here. Simon described only the male of *P. chlorophthalmus*, although the vial with a type specimen contains also an undescribed female and a few immature specimens. This material is however in a very poor condition.

Length of the apical part of embolus varies. It is very long in South African specimen, extending beyond the retrolateral edge of apical part of cymbium (Figs 161, 162), and short in specimen from Congo D. R. (Figs 163, 164).

#### Distribution.

Known from Congo and South Africa (Fig. 197).

### Peplometus congoensis sp. nov.

Taxon classificationAnimaliaAraneaeSalticidae

E6B3FE65-FF22-584A-9099-C6A292E81642

http://zoobank.org/41E2658D-9A97-49CA-923F-E8A3935F9795

Figures 133 , 172–176 , 177–183 , 198

#### Holotype.

Congo • ♂; Brazzaville, ORSTOM Park; 4°16'S, 15°17'E; 19.X.1963; J. Balogh and A. Zicsi leg.; HNHM.

#### Paratypes.

Congo • 1♀; together with the holotype. Congo D.R. • 1♀; Mayombe, Bas Congo, Luki Forest Reserve; 5°37'S, 13°05'E; 28.IX.2007; D. De Bakker and J.P. Michiel leg.; primary rainforest, canopy fogging; MRAC 226 108.

#### Diagnosis.

**Figures 172–176.**
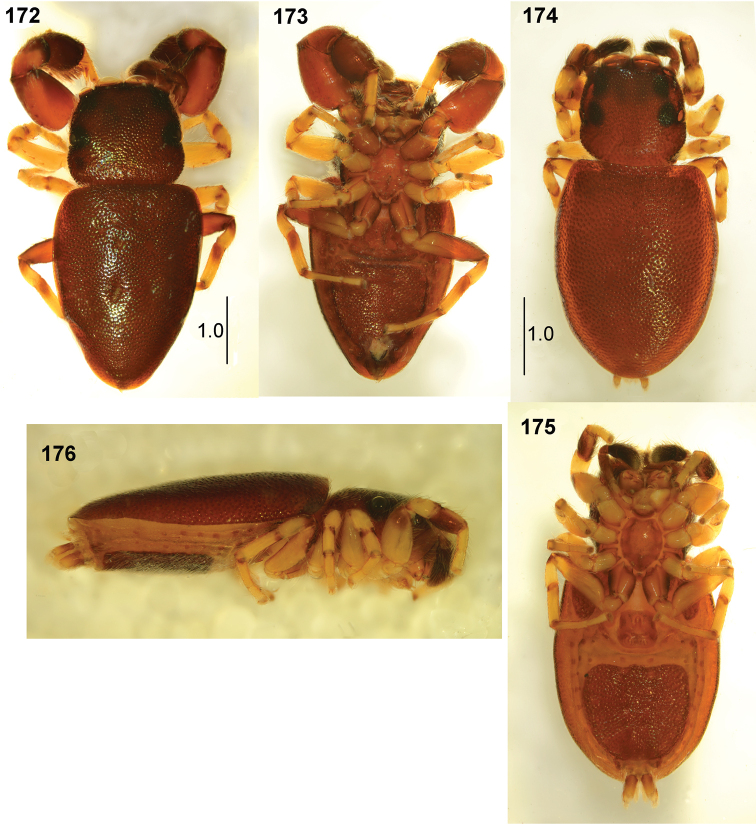
*Peplometus congoensis* sp. nov. **172** male, holotype, habitus, dorsal view **173** male, habitus, ventral view **174** female, habitus, dorsal view **175** female, habitus, ventral view **176** female, habitus, lateral view.

The most characteristic feature of this species is colouration and shape of the first pair of legs. Tibia I in male is totally black, it is not flattened (Fig. 172), whereas in congeners tibia is flattened dorsally or laterally and has a light streak or patch. Tibia I of the female is light retrolaterally, with large dark patch at its basis (Figs 179, 180). The abdomen of female is narrow, similar as in male, while females of other species have abdomen wider than males.

#### Etymology.

The species is named after its *terra typica* (Congo).

#### Description.

**Male.** Measurements. Cephalothorax: length 1.7, width 1.8, height 0.6. Eye field: length 0.9, anterior width 1.3, posterior width 1.7. Abdomen: length 3.0, width 2.2.

**Figures 177–183.**
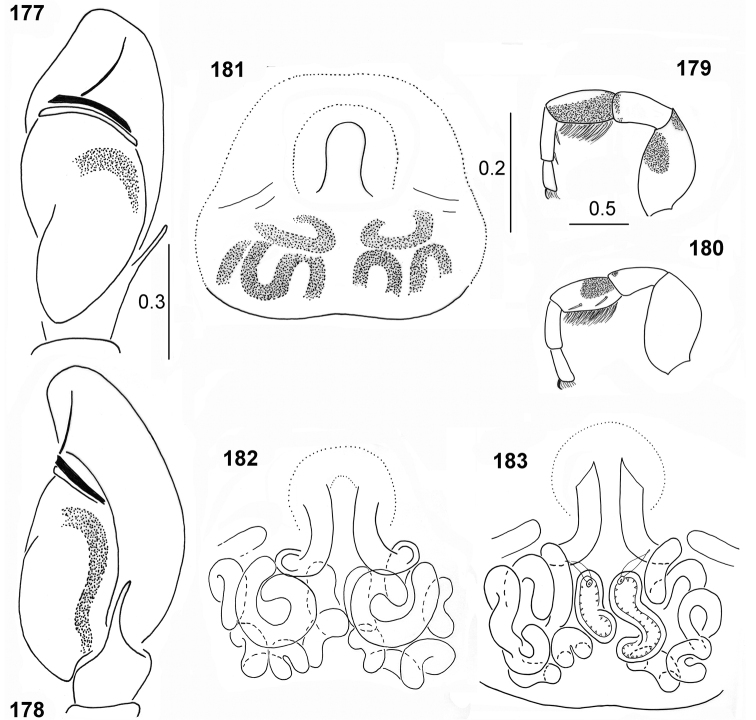
*Peplometus congoensis* sp. nov. **177** palpal organ, ventral view **178** palpal organ, lateral view **179** first leg of female, prolateral view **180** first leg of female, retolateral view **181** epigyne **182** internal structure of epigyne, ventral view **183** internal structure of epigyne, dorsal view.

General appearance as in Figs 172, 173. Body flattened, integument strongly sclerotised and clearly pitted. Carapace trapezoid, widest posteriorly, dark brown, black around eyes, a few bristles at eyes of first row. Chelicera with two small teeth on promargin and four-tip retromarginal tooth. Mouth parts and sternum light brown. Abdomen elongated, only slightly wider than carapace, venter also covered by scuta, posterior scutum large (Fig. 173). Legs I stout, brown, lateral side of femur slightly darker, tip of patella dark, tibia strongly thickened, black, with long dense black feather-shaped setae ventrally (Figs 133, 172). Other legs yellowish brown, with distal small dark brown patches on patellae and tibiae II–III and proximal brown rings on patellae, tibiae and metatarsi IV, femora IV brown. Palpal organ as in Figs 177, 178, apical part of embolus long.

**Female.** Measurements. Cephalothorax: length 1.2–1.3, width 1.1–1.3, height 0.4–0.5. Eye field: length 0.7, anterior width 1.0–1.1, posterior width 1.1–1.3. Abdomen: length 2.6–3.0, width 1.8–1.9.

General appearance as in Figs 174–176. Carapace rounded, brown to black, strongly sclerotised and clearly pitted, eye field on half of its length. Some dark bristles at eyes of first row. Chelicerae yellowish brown, with short fang, two small teeth on promargin and three-tip tooth on retromargin. Mouth parts creamy. Sternum oval, blackish. Abdomen relatively narrow, elongated, widest anteriorly, anterior edge almost straight. Dorsum of abdomen totally covered by very strongly sclerotised, pitted, black scutum, turned back on its margin. Abdomen ventrally with hard scuta; anterior scutum narrow along anterior margin of abdomen, extending backwards, posterior scutum large, trapezoid. Numerous bumps on sides of abdomen venter (Fig. 175). Spinnerets dark. Legs generally light, creamy with blackish stains. Legs I the biggest (not so markedly as in males); femora with black patch distally on prolateral side, tibia thickened, prolaterally black, basal half of retrolateral part black (Figs 179, 180). Dense long black setae on ventral side of tibia, two pairs of ventral spines. Dark line along dorsum of femora IV. Palps blackish. Epigyne large, rectangular, with small depression divided by median ridge, hardly visible horizontal crevices laterally (Fig. 181). Internal structure as in Figs 182, 183.

#### Distribution.

Known only from Congo and DR Congo (Fig. 198).

### Peplometus nimba sp. nov.

Taxon classificationAnimaliaAraneaeSalticidae

6680F74F-B260-5D35-A44A-4DA95706147B

http://zoobank.org/30CA27D6-8CC9-4960-B63C-9517D696F20A

**Figures 184–188.**
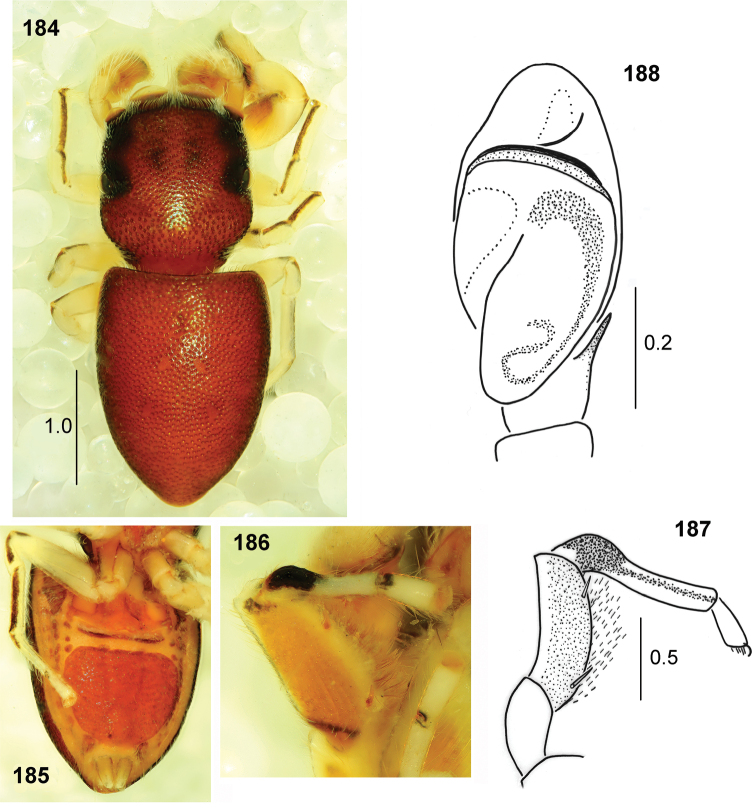
*Peplometus nimba***184** male, holotype, habitus, dorsal view **185** venter of abdomen **186** first leg, retrolateral view **187** first leg, prolateral view **188** palpal organ, ventral view.

Figures 135 , 184–188 , 198

#### Holotype.

Guinea • ♂; Nimba Mts, Nion; 7°36'N, 8°28'W; 16.VI.1942; M. Lamotte leg.; BMNH.

#### Diagnosis.

Male of this species is very similar to that of *Pachyballus oyo*, it can be distinguished by the lack of a conspicuous, sharp process on tibia I, which is very characteristic for the latter species (compare Fig. 135 with Fig. 134). In *P. nimba* metatarsus and tibia of leg I are of equal length, while in *P. oyo* metatarsus I is clearly shorter, about half of tibia I length.

#### Etymology.

The specific name is a noun in apposition referring to the Nimba Mts, type locality of this species.

#### Description.

**Male.** Measurements. Cephalothorax: length 1.4, width1.2, height 0.5. Eye field: length 0.7, anterior width 1.1, posterior width 1.2. Abdomen: length 2.0, width 1.4.

General appearance as in Fig. 184. Body very flat, covered with strongly sclerotised integument, clearly pitted, brown with blackish area around eyes. Carapace slightly trapezoid, white bristles near eyes of first row. Clypeus very low. Chelicerae with two teeth on promargin and five on retromargin (apical tooth very small), fang short. Endites, labium and sternum yellow. Abdomen elongated, shield shaped (its anterior margin almost straight), dorsum covered with large strongly sclerotised scutum. Ventrally abdomen with typical large scuta (Fig. 185), as in other species. In front of epigastric furrow clearly visible narrow wedge-shaped sclerotised swelling. Spinnerets yellow. First pair the stoutest, femora basically yellow, slightly darker on sides; patella light yellow, tibia slightly thickened, brown (prolateral surface darker), with light streak dorsally, dense long whitish (probably bleached) setae ventrally; metatarsus long, creamy with black dorsal hump and black line along prolateral side (Fig. 187). Legs II–IV yellow, with thin dark line along prolateral sides of patellae and tibiae. Legs without spines, except two short ventral spines on metatarsus I. Palps yellow, tibial apophysis very thin, bulb triangular, embolus spirally coiled on bulb tip (Fig. 188).

**Female** unknown.

#### Distribution.

Known only from the type locality, Nimba Mts in western Africa (Fig. 198).

### Peplometus oyo

Taxon classificationAnimaliaAraneaeSalticidae

(Wesołowska & Russell-Smith, 2011) comb. nov.

A1AA4D27-5DEC-5792-A0BD-778E45AAE0BA

Figures 134 , 198

Pachyballus oyo Wesołowska and Russell-Smith 2011: 588, f. 126–135, 232–234 (♂♀).

#### Diagnosis.

The male of this species is similar to that of *Peplometus nimba*, but it can be identified by the presence of a big apical process on the first pair of legs and the metatarsus of this pair shorter than tibia (Fig. 134).

#### Description.

For description of both sexes see: Wesołowska and Russell-Smith (2011).

#### Distribution.

Southern Nigeria (Fig. 198).

**Figures 189–192.**
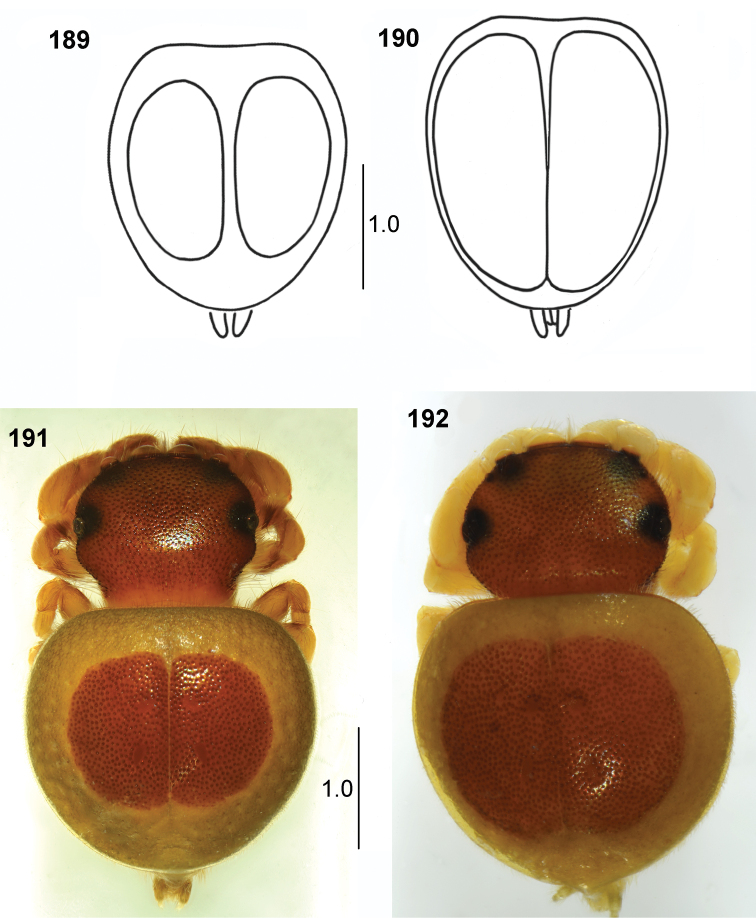
Dorsal scuta of abdomen, immature specimens. **189, 190***Peplometus biscutellatus***191, 192***Pachyballus transversus***189, 191** younger instar **190, 192** subadult.

**Figures 193–198.**
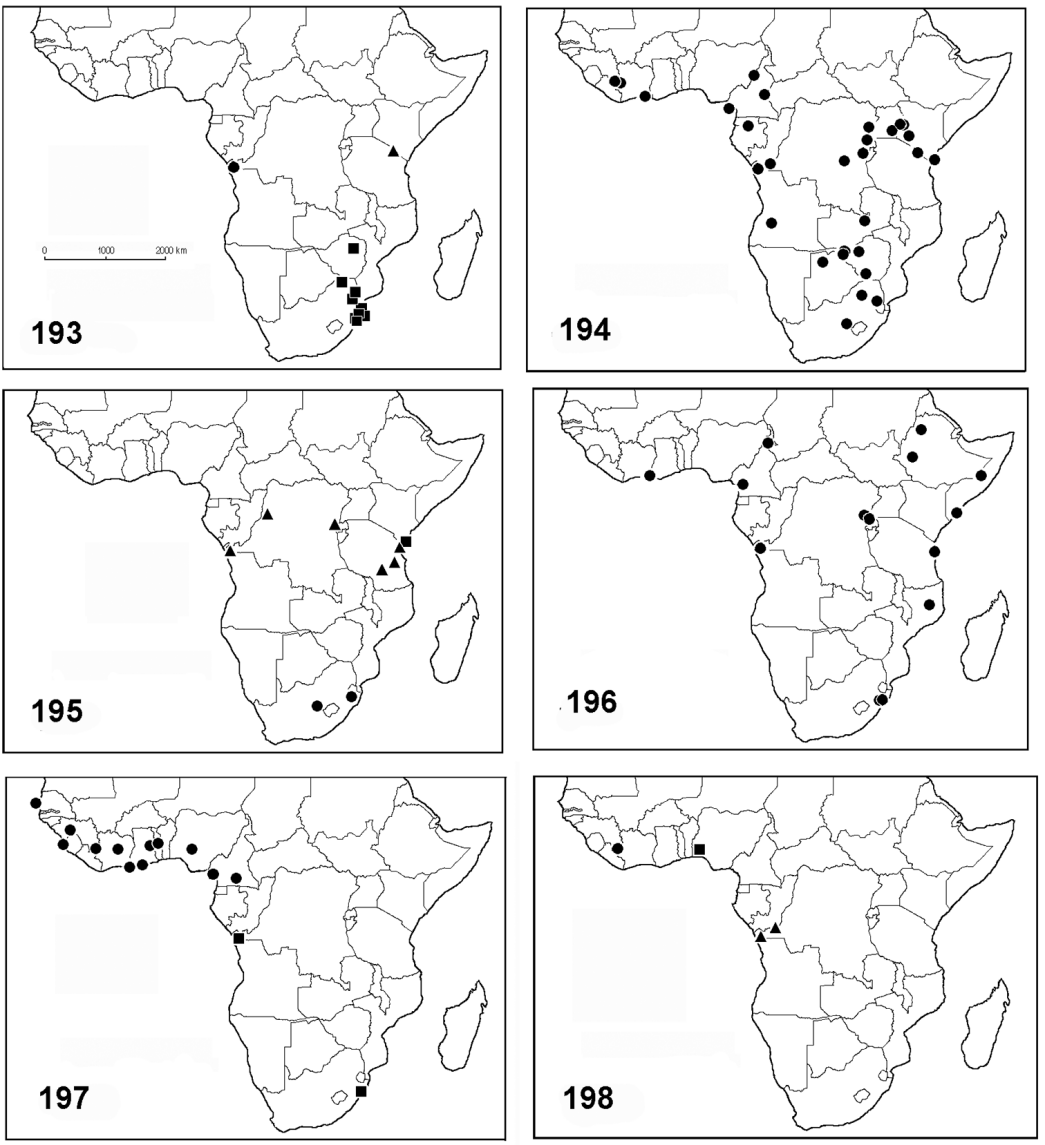
Distribution maps of *Pachyballus* and *Peplometus* species. **193** circle – *Pachyballus caelestis*, square – *P. castaneus*, triangle – *P. variegatus***194***P. flavipes***195** circle – *P. miniscutulus*, square – *P. mombasensis*, triangle – *P. ornatus***196***P. transversus***197** circle – *Peplometus biscutellatus*, square – *P. chlorophthalmus***198** circle – *P. nimba*, square – *P. oyo*, triangle – *P. congoensis*.

## Mimicry

The mimicry is a common phenomenon among spiders (Pocock 1909). Jumping spiders imitate various models, mainly ants (overview in Cushing 1997, 2012), but also pseudoscorpions (Platnick 1984), flies (Morrison 1981), wasps (Reiskind 1976, Żabka 1992), velvet wasps (Edwards 1984, Wesołowska 2009) or caterpillars (Logunov and Obenauer 2019). Some salticid genera resemble beetles (Cloudsley-Thompson 1995) e.g. *Cylistella* Simon, 1901, *Coccorchestes* Thorell, 1881, *Planiemen* Wesołowska and van Harten, 2007. The Ballini genera *Pachyballus* Simon, 1901 and *Peplometus* Simon, 1901 also resemble beetles, most probably from the family Chrysomelidae, which is expressed in their body shape and a very strongly sclerotised integument. The microsculpture of the integument and bright or iridescent colouring emphasise this resemblance. In juveniles of all *Peplometus* species and *Pachyballus transversus* the two dorsal scuta on the abdomen are arranged in a pattern similar to elytra of beetles (Figs 165, 189–192), it is the unique feature among spiders. This type of resemblance in spiders usually suggests a Batesian type of mimicry. On the other hand, the two presented genera live in the canopy and their body type (small, flattened, colours often matching their preferred habitats) may suggest that it is a type of camouflage. The resemblance to other tree-living arthropods could be just a result of a shared cryptic body pattern.

## Supplementary Material

XML Treatment for Pachyballus

XML Treatment for Pachyballus caelestis

XML Treatment for Pachyballus castaneus

XML Treatment for Pachyballus flavipes

XML Treatment for Pachyballus gambeyi

XML Treatment for Pachyballus miniscutulus

XML Treatment for Pachyballus mombasensis

XML Treatment for Pachyballus ornatus

XML Treatment for Pachyballus transversus

XML Treatment for Pachyballus variegatus

XML Treatment for Peplometus

XML Treatment for Peplometus biscutellatus

XML Treatment for Peplometus chlorophthalmus

XML Treatment for Peplometus congoensis

XML Treatment for Peplometus nimba

XML Treatment for Peplometus oyo
